# The effect of *Oenothera biennis* (Evening primrose) oil on inflammatory diseases: a systematic review of clinical trials

**DOI:** 10.1186/s12906-024-04378-5

**Published:** 2024-02-15

**Authors:** Melika Sharifi, Nasim Nourani, Sarvin Sanaie, Sanaz Hamedeyazdan

**Affiliations:** 1 Student Research Committee, Faculty of Pharmacy, Tabriz University of Medical Sciences, Tabriz, Iran; 2 Department of Pharmaceutical Chemistry, Faculty of Pharmacy, Tabriz University of Medical Sciences, Tabriz, Iran; 3 Research Center for Integrative Medicine in Aging, Aging Research Institute, Tabriz University of Medical Sciences, Tabriz, Iran; 4 Department of Pharmacognosy, Faculty of Pharmacy, Tabriz University of Medical Sciences, Tabriz, Iran

**Keywords:** *Oenothera biennis*, Evening primrose oil, Inflammation, Gamma-linolenic acid, Systematic review

## Abstract

**Background:**

Evening primrose oil (EPO), extracted from the seeds of *Oenothera biennis*, has gained attention for its therapeutic effects in various inflammatory conditions.

**Method:**

We performed a systematic search in multiple databases and defined the inclusion criteria based on the following PICOs: P: Patients with a form of inflammatory condition, I: EPO, C: Placebo or other therapeutic interventions, O: changes in inflammatory markers or patients’ symptoms; S: randomized controlled trials. The quality of the RCTs was evaluated using Cochrane’s RoB tool.

**Results:**

Several conditions were investigated in the literature. In rheumatoid arthritis, mixed results were observed, with some studies reporting significant improvements in symptoms while others found no significant impact. EPO showed some results in diabetes mellitus, atopic eczema, menopausal hot flashes, and mastalgia. However, it did not demonstrate effectiveness in chronic hand dermatitis, tardive dyskinesia, psoriatic arthritis, cystic fibrosis, hepatitis B, premenstrual syndrome, contact lens-associated dry eyes, acne vulgaris, breast cyst, pre-eclampsia, psoriasis, or primary Sjogren's syndrome. Some results were reported from multiple sclerosis after EPO consumption. Studies in healthy volunteers indicated no significant effect of EPO on epidermal atrophy, nevertheless, positive effects on the skin regarding hydration and barrier function were achieved.

**Conclusion:**

Some evidence regarding the potential benefits of EPO in inflammatory disorders were reported however caution is due to the limitations of the current survey. Overall, contemporary literature is highly heterogeneous and fails to provide strong recommendations regarding the efficacy of EPO on inflammatory disorders. Further high-quality studies are necessitated to draw more definite conclusions and establish *O. biennis* oil effectiveness as an assuring treatment option in alleviating inflammatory conditions.

**Supplementary Information:**

The online version contains supplementary material available at 10.1186/s12906-024-04378-5.

## Introduction

Inflammatory diseases, characterized by chronic inflammation and immune dysregulation, continue to pose significant challenges in healthcare, with their prevalence and impact on global health steadily increasing. Inflammation is a complex biological response that plays a crucial role in the pathogenesis of numerous diseases, including rheumatoid arthritis (RA), diabetes mellitus (DM), atopic eczema (AE), and many others [1–3]. Conventional therapies for inflammatory diseases often involve the use of nonsteroidal anti-inflammatory drugs (NSAIDs), corticosteroids, immunosuppressants, or biologic agents. However, these treatments may be associated with adverse effects, limited efficacy, or high costs, necessitating exploring of alternative therapeutic options. Traditional medicine, rooted in centuries-old practices and knowledge systems and including the use of herbal remedies, has emerged as a valuable resource in the management of inflammatory diseases [4, 5]. As far as we know, synthetic and semi-synthetic pharmaceutical derivatives from plants are being used in most clinical drugs and with the spread of various diseases, hundreds of plant-based molecules are ongoing to be discerned and discovered [6]. Given that, natural compounds have also been used in the anti-inflammatory diseases [7, 8]. Evening primrose oil (EPO), derived from the seeds of *Oenothera biennis*, has gained attention for its potential therapeutic effects in various inflammatory diseases [9]. EPO is rich in essential fatty acids(EFAs), including linoleic acid (LA) and gamma-linolenic acid (GLA), which is a precursor for anti-inflammatory substances in the body (6) [9]. GLA is metabolized into prostaglandin E1 (PGE1), a potent anti-inflammatory mediator that can modulate immune responses and reduce inflammation. Additionally, EPO contains other bioactive compounds, such as flavonoids and phenolics, which possess antioxidant and anti-inflammatory properties. The identified polyphenols and flavonoids is previous studies were mainly gallic acid, caffeic acid, epicatechin, coumaric acid, ferulic acid, rutin and rosmarinic acid [9]. In another study again, ( +)-catechin, (-)-epicatechin and gallic acid were reported to be the main components of EPO phenolic compounds [10] and one isoflavone together with 2-hydroxychalcone were revealed in EPO [11].

Lipoxygenase (LOX) and cyclooxygenase (COX) are the two pro-inflammatory enzymes that synthesize the eicosanoids (like leukotriene (LT), prostaglandin (PG)) from arachidonic acid (AA), and therefore, playing an essential role in inflammatory processes. Based on aforementioned statement, EPO components, showed anti-inflammatory activities via inhibition of LOX [3, 12]. It is reported that EPO was beneficial in different types of inflammatory diseases. To assess the potential benefits of EPO in inflammatory diseases, a systematic review of relevant clinical trials was conducted. The primary objective was to synthesize the available evidence and critically evaluate the effectiveness of EPO in improving symptoms, reducing disease activity, and enhancing overall outcomes in inflammatory conditions.

### Methodology

The current literature protocol is registered in the International Register of Prospective Systematic Reviews (PROSPERO ID: CRD42023394200). This systematic review and meta-analysis was conducted using the accepted systematic review method of the book entitled "A Systematic Review for Evidence-Based Support Medicine" [13] and according to the Preferred Reporting Items for Systematic Reviews and Meta-Analyses (PRISMA) [14].

### Information sources and search strategy

As shown in Table 1, proper search terms were defined based on the PICOs and combined using Boolean operators to produce each search string. The following databases were searched: Google Scholar, PubMed, EMBASE, Scopus, and Cochrane Central Register of Controlled Trials. The search was carried out by two independent reviewers in the end of December 2022. Some of the applicable journals and websites were also searched manually. Reference lists of the chosen articles were reviewed as well.

**Table 1:** Different search terms and combinations used in designing the search strategy based on PICOs^a^

Population/patients	Intervention	Outcome	Study design
Inflammatory disease	*Oenothera biennis*	Inflammation marker	Randomized controlled trial
Inflammation	*Oenothera biennis*	Disease activity	Randomized clinical trial
Inflammation	Evening Primrose	Pain	RCT
Autoimmune disease	Evening, Primrose, Oil	Quality of life	Clinical Trials, Randomized
Rheumatoid arthritis	Onagraceae	Adverse effects	Trials, Randomized Clinical
Cardiovascular disease	*Oenothera biennis*	Cytokine level	Controlled Clinical Trials, Randomized
high blood pressure		Symptom improvement	
Gastrointestinal diseases		Skin condition	
Inflammatory bowel disease		Gut inflammation	
obstructive pulmonary disease			
COPD			
Asthma			
Metabolic disease			
Diabetes Mellitus			
Psoriasis			
Eczema			
Atopic dermatitis			

^a^The *OR* Boolean operator was used between the terms in each column, while *AND* was used to combine the columns

### The inclusion and exclusion criteria

The inclusion and exclusion criteria were defined in accordance to the PICOs, which is defined in the Table 1.

Inclusion criteria are as follows:

- Studies with adult participants (≥ 18 years) suffering from an inflammatory disease of any nature, including rheumatologic, gastrointestinal, cardiovascular, metabolic, etc.
- Studies using Oenothera biennis (Evening primrose) oil as an intervention.
- Randomized controlled trials (RCTs).
- Published in English.

Exclusion criteria were:

- Using any other form of intervention in the absence of evening primrose oil
- Animal studies and basic experiments.
- Unoriginal publications, reviews, overviews, letters, summaries of meetings, etc.
- Unpublished or duplicate literature.
- Unavailable full text.

### Study selection

Two impartial reviewers (M.SH and N.N) reviewed the publications by reading the titles and abstracts after eliminating duplicate research. Next, the full texts of papers pertinent to the study's aims were carefully studied to establish eligibility. Disagreements about study selection were resolved by discussions between two researchers. In case of disagreement, it was referred to the third reviewer (S.S).

### Data extraction and items

Further, the data from each finalized paper was extracted into a predefined Tables 2 and 3. The following data was extracted from each study: country, author, gender, age, type of study, outcome/side effect, inflammatory factor, dosage, type of administration, period, patient number in intervention groups and control, disease duration, and time of assessment/base treatment regimen (Tables 2 and 3). The data was retrieved independently by two reviewers who were well-versed in the matter. Any disagreements were resolved by discussion or referring to a third reviewer.

**Table 2:** Characteristics of included clinical trial studies in the systematic review of EPO effectiveness (orally administered) on inflammatory diseases

No.	Author	Type and duration of disease	Number of participants			Female/Male ratio			Age		Type of intervention and dosage	
			Intervention	Control		Intervention	Control		Intervention	Control	Intervention	Control
1	Tomic-Smiljanic [17]	RA 59 months, SD ± 60 (12–180 months	20	20 in fish oil 20 placebo (only previous described rheumatologic therapy in the period of 3 months)		20/0	30/0		57,3 ± 8: group 2	Group 1: 54 ± 8 Group 3: 59 ± 7.5	daily after meals 2 gel capsules Omega-3 Cardio® and 2 gel capsules EPO each cap = 1300mg EPO (LOQ = 2600 mg/day)	daily after meals 5000 mg of omega-3 PUFA (5 gel capsules Omega-3 Cardio®/
2	BELCH [18]	RA 5 YEARS	31 (16 patients: EPO, 15: EPO/fish oil)	18		EPO: 15/1 EPO/FO: 11/4	17/1		EPO: 46 (35–68) EPO/FO: 53 (28–73)	48 (30–74)	3 cap 4 times a day (540 mg GLA/ 450 mg GLA + 240 mg EPA) (LOQ = 2600 mg/day)	3 cap 4 times a day (liquid paraffin)
3	BRZESKI [19]	RA 8 YEARS	19	21		17/2	15/6		60 (54–77)	61 (51–67)	6g/day (EPO) (LOQ = 6000 mg/day)	6g/day (olive oil)
4	JANTTI [20]	RA / The duration of RA was 13 years in the EPO group and 10 years in the olive oil group	10	10		9/1	9/1		50	38	10 ml twice daily (EPO) (LOQ = 20 ml/day)	10 ml twice daily (olive oil)
5	Veselinovic [21]	RA/59 ± 60 month (12–180 months)	40 (20:FO/ 20: EPO + OMEGA)	20		40/0 (Group 1 = group 2 = 20 patients)	20/0		Group1: 54 ± 8/ group 2: 57 ± 8	59 ± 7	5g fish oil (5g)/ 2 omega cap + 2 EPO (2600mg) cap after meal (LOQ = 2600 mg/day)	described rheumatologic therapy
6	Jamal [22]	Diabetic Peripheral/ minimum duration of symptoms of neuropathy was 6 months and of diabetes 3 years	12	10		7/5	5/5		53 ± 19 (21–74)	55 ± 15 (23–74)	4 cap twice daily (8 cap:4g) (LOQ = 4000 mg/day)	4 cap twice daily (8 cap:4g)
7	Arisaka [23]	Diabetes Mellitus//5–5.3 years	6 (EPO)	5 (indistinguishable placebo capsule)		6/5			12 years	13 years	2 capsules (each cap = 360 mg of LA and 45 mg of GLA) daily for 4 months then 4 capsules daily for a further 4 months (LOQ = 1000 mg/day)	2 daily for 4 months then 4 capsules daily for a further 4 months
8	Bamford [24]	AE	123						49children (ages 2 to 16 years; mean, 9.1) and 74 adults (ages 16 to 66 years; mean, 37.7)		Children (subjects 15 years old or younger) received two or four capsules twice daily, and adults received six or eight capsules twice daily Each cap = 500 mg EPO (LOQ = 1000 mg/day)	Children (subjects 15 years old or younger) received two or four capsules twice daily, and adults received six or eight capsules twice daily Each cap = 500 mg Liquid paraffin
9	MANKU [25]	AE/ history beginning in childhood, together with a personal and/or family history of other atopic disorders	41 (A → 16 patients, B → 13, C → 12)	50			18/32		mean age 24 years		2, 4, and 6 g/day (A,B,C) EPO (LOQ = 2000 mg/day)	
10	SCHALIN-KARRILA	AE	14	11		16/9			19 to 31 years		Four capsules twice daily (EPO) Each cap = 360 mg linoteic acid, 50 mg oleic acid and 45 mg GLA (LOQ = 4000 mg/day)	Four capsules twice daily Each cap = 500 mg of liquid paraffin
11	WRIGHT [26]	AE (every patient also had either a family history of atopy or a personal history of other atopic symptoms. In every patient the disease was moderate or severe)	81	18					60 adults aged 15–58 years and 39 children aged 8 months to 14 years		Group A: two capsules twice daily, group B: four capsules twice daily, group C: six capsules twice daily. In the children’s group, 20 received one capsule twice daily (group D) Each cap = 360 mg of LA and 45 mg of GLA (LOQ = 500 mg/day)	two capsules twice daily (group E) each cap = liquid paraffin: 500mg
12	Breth-jones [27]	Atopic dermatitis	133 were enrolled								6 cap twice/day (500 mg EPO/ 430 mg EPO + 107 mg fish oil (LOQ = 6000 mg/day)	6 cap twice/day (liquid paraffin: adults olive oil: children)
13	Whitaker [28])	Chronic Hand Dermatitis /More than 1 year	20	19					19–75 years		12 cap/day (each cap = 500 mg Epogam) (LOQ = 6000 mg/day)	12 cap/ day (each cap = 500 mg sunflower oil)
14	EBDEN [29]	Atopic Asthma	12			8/4			mean age: 33 years (range 20–52)		two Efamol capsules four times daily (360 mg of LA and 45 mg GLA) (LOQ = 4000 mg/day)	two capsules four times daily (500 mg of liquid paraffin)
15	Hederos [30]	atopic dermatitis (epogam:0.9 years/placebo: 1.6 years) and asthma	eczema			eczema			eczema		1–12 years: 4 capsules twice daily/ over 12 years: 6 capsules twice daily → each cap = 500 mg EPO: 40 mg GLA + 10 mg vit E (LOQ = 4000 mg/day)	1–12 years: 4 capsules twice daily/ over 12 years: 6 capsules twice daily → Each cap = 500 mg sunflower oil + 10 mg vit E
			30	30		17/13	17/13		7.5 (1–14)	8.6 (2–16)		
			asthma			asthma			asthma			
			12	10		5/7	4/6		9.3 (4–14)	10.9 (3–16)		
16	Blommers [31]	severe chronic mastalgia/ cyclic or noncyclic mastalgia for > 6 months, (2) an average of ≥ 7 and a minimum of 5 days with breast pain per menstrual cycle	90 (each group 30)	30		90/0	30/0		36.8 ± 6.2	22	3g/day (Group FC: fish oil and control oil, group EC: EPO and control oil, group EF: fish oil + EPO) (LOQ = 3000 mg/day)	(group CC: two control oils)
17	Goyal [32]	Mastalgia average length 23–33 days), moderate to severe mastalgia of a minimum of 3-months duration requiring drug treatment, with at least 7 days of pain per menstrual cycle	417	138		280/0	275/0		GLA + multivitamines: 39.6 (6.8) GLA + multivitamines: 39.7 (6.0)	Placebo + multivitamines: 39.2 (6.5) Both placebo: 39.0 (6.6)	1 Cap/day (a:500 mg EPO (40 mg GLA) and 10 mg vit E. b: 500 mg coconut oil + 10 mg vit E. c:3 mg beta carotene, 100 mg vitamin C, 25 mg vitamin B6, 10 mg zinc, 10 mg niacin and 455 µg selenium d: 255 mg fractionated coconut oil → ((a) GLA and antioxidant/(c) GLA and placebo antioxidants (LOQ = 500 mg/day)	(b) placebo fatty acids and antioxidants/(d) placebo fatty acids and placebo antioxidants
18	Pye [33]	Mastalgia/ for at least 6 months, for a minimum of 10 days in each cycle				/0	/0				6 cap/day	
19	Qureshi [34]	Mastalgia/moderate to severe breast pain of two to three months duration over a period of one year	25	25		25/0	25/0		15 to 50 years		500mg twice daily OEP capsules (Efamol) (LOQ = 1000 mg/day)	application to the affected area twice a day topical NSAID in 0.5% Piroxicam gel (Feldene
20	Nasri [35]	polycystic ovary syndrom	30	30		30/0	30/0		18–40 years		1000 IU vitamin D3 plus 1000 mg EPO (LOQ = 1000 mg/day)	placebo
21	Farzaneh [36]	menopausal hot flashes mean duration of menopause was 2.4 ± 1.8 (range: 1–7) years	56			56/0			45–59 years		two capsules per day = 1g/day (totally 90 capsules for 6 weeks) (LOQ = 1000 mg/day)	Placebo:1g/day
22	Gateley [37]	benign breast disorders /mastalgia	36			36/0					8 capsule (320 mg GLA)s daily (EPO) (LOQ = 4000 mg/day)	8 capsules liquid paraffin
23	Gateley [37]	Breast cyst	200			200/0					six capsules daily (240 mg GLA) (LOQ = 1500 mg/day)	six capsules daily placebo
24	Gupta	Hypercholesterolemia and Mixed Dyslipidemia	30	30		21/9	22/8		46.80 ± 7.43 years	45.50 ± 6.76 years	1 cap/day after 2 main meal (250 mg EPO) (LOQ = 250 mg/day)	1 cap/day after 2 main meal placebo
25	Ishikawa [38]	hypercholesterolemic	19			7/12			42–63 years		3.6g/day (four capsules containing 0.3 g of EP three times daily,) (LOQ = 3600 mg/day)	3.6g/day (four capsules containing 0.3 g of sunflower oil three times daily)
26	JENKINS [39]	chronic hepatitis B/ presence of hepatitis B surface antigen in two serum samples at least 6 months apart	10 (11 entered)	10 (13 entered)		1/9	2/8		59.4 ± 9.9	45.6 ± 13.5	4g/day (2g twice daily before meal( each cap: 500 mg + 10 mg Vit E) (LOQ = 4000 mg/day)	4 capsules (2 g) twice daily before meals liquid paraffin:
27	Khoo [40]	PMS	19	19		19/0	19/0		20–40 years			
28	Kokke [41]	lens associated dry eye/ wearing monthly or daily soft contact lenses	28	24		28/0	24/0		46.4 (12.6)	37.3 (10.7)	6 cap/day (EPO)	6 cap/day Olive oil
			76 entered									
29	Laivuori [42]	Pre-eclamptic	7 (EPO:4, FO:3)	5		7/0	5/0		EPO: 32.0 (23–40), FO: 30.3 (24–40)	30.2 (26–32)	10 g/day (EPO, fish oil) (LOQ = 10,000 mg/day)	Olive oil (Each cap: 500 mg of maize oil and 500 mg of olive oil)
			18 entered (primrose oil (n = 7), with fish oil (n = S), or with placebo (n = 6))									
30	MOODLEY	Pre-Eclampsia /32–36 years	23	24		23/0	24/0		(17–27)	( 16–27)	8 cap/day (each cap: 500 mg EPO) (LOQ = 4000 mg/day)	8 cap/day
31	Makrides [43]	Erythrocyte fatty acid changes of term infants	13	32		9/4	26/17				(FO + EPO) one sachet to 200 mL (LOQ = 200 ml/day)	placebo powder to 210 mL
				19 PLACEBO	23 fully breast feed		11/8	14/9				
32	Manthorpe [44]	Primary Sjogren's syndrome/ 1–40 YEARS: FEMALE, 2–3 YEARS: MALES	36			33/3			34–76 YEARS		three capsules of Efamol twice daily and three tablets of Efavit twice daily (LOQ = 3000 mg/day)	three tablets of placebo
33	OLIWIECKI [45]	Primary Sjogren's syndrome	37						16–70 years		12 cap/day (2 divided dose) Each cap = (430 mg EPO) (LOQ = 6000 mg/day)	12 cap/day (2 divided dose) Each cap = 500 mg Liquid paraffin
34	Theander	Primary Sjogren's syndrome/ 6–14 years (10)	87			79/8			50–68 years (62)		EPO: 800 mg or 1600 mg per day Each cap = 40 or 80 mg (2 different dose in 2 groups) (LOQ = 800 mg/day)	1600 mg per day Corn oil emulsion
35	Ka ´zmierska [46]	Acne Vulgaris	25	25					18 to 30 years (mean age 22.0 ± 2.07 years)		4 cap/day (each:510 mg/ 2 morning and 2 evening (LOQ = 2000 mg/day)	4 cap/day (each = 10 to 40 mg of isotretinoin
									22.5 ± 1.92 years	21.6 ± 2.14 years		
36	Oxholm [47]	Primary Sjogren's syndrome/ 7 years (range 1–23)	28			24/4			Mean age was 51 years (range 32–71)		one period of 8 weeks with 3 g Efamol daily (6 capsules) (LOQ = 3000 mg/day)	another period of 8 weeks with identical looking placebo capsules
37	Iran/Soheila Rezapour-Firouzi a,b, ∗ , Seyed Rafie Arefhosseini	multiple sclerosis/A: 6.26 ± 3.9/ B: 7.55 ± 5.08/ C: 6.60 ± 4.0	43 (A:23, C:20)	22		12/31 (A: 7/16, C: 5/15)	11/11		A: 34.2 ± 7.5 C: 33.7 ± 7.8	35.9 ± 7.8	18—21 g/day (6—7 g, three times daily) (‘‘Group A’’ received co-supplemented hemp seed and evening primrose oils with advised Hot-nature diet (LOQ = 1800 mg/day)	‘‘Group B’’ who received olive oil, ‘‘Group C’’ who received the co-supplemented oils)
38	Rezapour-Firouzi [48]	multiple sclerosis/A: 6.26 ± 3.9/ B: 7.55 ± 5.08/ C: 6.60 ± 4.0	43 (A:23, C:20)	22		12/31 (A: 7/16, C: 5/15)	11/11		A: 34.2 ± 7.5 C: 33.7 ± 7.8	35.9 ± 7.8	18—21 g/day (6—7 g, three times daily) (‘‘Group A’’ received co-supplemented hemp seed and EPO with advised Hot-nature diet (LOQ = 1800 mg/day)	‘‘Group B’’ who received olive oil, ‘‘Group C’’ who received the co-supplemented oils)
39	Rezapour-Firouzi [49]	multiple sclerosis/A: 6.26 ± 3.9/ B: 7.55 ± 5.08/ C: 6.60 ± 4.0	43 (A:23, C:20)	22		12/31 (A: 7/16, C: 5/15)	11/11		A: 34.2 ± 7.5 C: 33.7 ± 7.8	35.9 ± 7.8	18—21 g/day (6—7 g, three times daily) (‘‘Group A’’ received co-supplemented hemp seed and EPOs with advised Hot-nature diet (LOQ = 1800 mg/day)	‘‘Group B’’ who received olive oil, ‘‘Group C’’ who received the co-supplemented oils)
40	Rezapour-Firouzi [50]	multiple sclerosis/A: 6.26 ± 3.9/ B: 7.55 ± 5.08/ C: 6.60 ± 4.0	43 (A:23, C:20)	22		12/31 (A: 7/16, C: 5/15)	11/11		A: 34.2 ± 7.5 C: 33.7 ± 7.8	35.9 ± 7.8	18—21 g/day (6—7 g, three times daily) (‘‘Group A’’ received co-supplemented hemp seed and evening primrose oils with advised Hot-nature diet (LOQ = 1800 mg/day)	‘‘Group B’’ who received olive oil, ‘‘Group C’’ who received the co-supplemented oils)
41	Vaddadi [51]	Tardive Dyskinesia	21	17		9/11	7/10		mean age of 52.7 years		12 capsules of Efamol in divided doses (LOQ = 6000 mg/day)	12 capsules of placebo in divided doses
42	VEALE [52]	Psoriatic arthritis/ 1–30 years	19	19		12/7	12/7		18–76): 40	(25–58): 40	12 capsules of Efamol daily Each cal = 480 mg GLA, 240 mg EPA and 132 mg of DHA (LOQ = 6000 mg/day)	12 capsules of placebo daily

No.	Author	Duration of intervention	Monitored inflammatory factors			Outcome	side effect
			diagnosis	Endpoint	Time point of blood samples		
1	Tomic-Smiljanic [17]	3 months	CRP → group 1: 12.4 ± 8.2 Group2: 16.0 ± 18.3 group3: 12.7 ± 7.2 ESR → group1: 35 ± 24 Group2: 36.7 ± 19.2 Group3: 33.25 ± 17.14			There was no significant difference in neither ADP nor arachidonic acid-induced platelet aggregation between the groups of patients with RA who used omega-3 PUFA and the patients with RA who used omega-3 PUFA and EPO	Mild gastrointestinal distress (mild diarrhea, abdominal pain, indigestion or nausea less than 72 h)
2	BELCH [18]	15 months	ESR → EPO: 4–81 (22) EPO/FO: 4–55 (26) placebo: 3–75 (30) CRP → EPO: 10–43 (19), EPO/FO: 10–38 (13), placebo: 8–76 (19)			94% of the EPO and 93% of the EPO/fish oil group felt a subjective improvement in their condition at 12 months. Most patients on active treatment recorded an increase in their general sense of wellbeing. In this study we have shown that it was possible for some patients with RA to decrease or stop NSAID treatment when EPO or EPO/fish oil was given. despite the lack of objective improvement in symptoms on the active oils, there was a very definite subjective improvement. The mechanism of this is unclear, but two alternative explanations are possible	nausea, diarrhoea, headache
3	BRZESKI [19]	6 months	ESR → EPO: 19–59 (41) placebo: 11–69 (42) CRP → EPO:10–45 (14), placebo: 10–53 (13)			No patients stopped NSAIDs but three in each group reduced the dose of NSAID—in all patients this was by only one tablet, e.g. ibuprofen 400 mg 3 times to 2 times a day—and one patient in the EPO group increased NSAID dosage. Four patients taking placebo and one taking EPO reduced analgesia dosage, and two in each group increased dosage. EPO produced marked reduction in morning stiffness and articular index, although only the former reached statistical significance	
4	JANTTI [20]	12 weeks	APO-A1 (g/l → EPO:1–366 (0.158), placebo: 0–965 (0–089) APO-A2 (g/l) → EPO: 1–445 (0–237), placebo: 0–89 (0–227)	APO-A1 (g/l → EPO: 1–330 (0–197), placebo: 1–188 (0–08) APO-A2 (g/l) → EPO: 1–149 (0–259), placebo: 0–752 (0–133)		Serum total cholesterol and triglyceride concentrations did not change in either of the groups, but the serum HDL-cholesterol concentration increased slightly during olive oil treatment. EPO had no effect on the serum concentration of apolipoprotein A-I, whereas that of apolipoprotein B decreased in all three patients studied. Apolipoprotein A-I increased in all four studied patients treated with olive oil, whereas apolipoprotein B decreased in three patients	
5	Veselinovic [21]	12 weeks	ESR → group1: 35.0 ± 24.1 Group 2: 36.7 ± 19.2, group3: 33.3 ± 17.1 CRP → group1: 12.4 ± 8.2, group2: 16.0 ± 18.3, group3: 12.7 ± 7.2	ESR → group1: 23.2 ± 16.6, group2: 19.9 ± 10.8, group3: 24.1 ± 13.9, CRP → group1: 7.3 ± 2.9, group2: 7.1 ± 5.5, group3: 6.9 ± 3.5		The number of painful joints and VAS score in both supplement groups (p ≤ 0.001) decreased significantly after 12 weeks, but not in the control group, a significant decrease in DAS 28 score was observed in the second group, which is n-3 have consumed PUFA. and EPO (4.76 ± 0.85 to 3.79 ± 0.72, group II). After 12 weeks of supplementation, when all groups were compared, the levels of EPA, DHA and n-3 PUFA were higher, and the ratio of n-6 to n-3 in both supplement groups was lower than that of control patients. GLA and AA were more in group II (fish oil + EPO) than groups I and III. The inflammatory factors decreased at the end of trial	Mild gastrointestinal discomfort (mild diarrhea, abdominal pain, dyspepsia or nausea lasting less than 72h
6	Jamal [22]	6 months	HbA1 (%) → EPO: 9.1 ± 0.4, Placebo: 8.8 ± 0.3	HbA1 (%) → EPO: 8.7 t 0.3, placebo: 8.9 ± 0.4		There was no significant change in the HbA, in either the active or the placebo group at the beginning or end of the trial period. At the end of the 6 months, no significant changes occurred in the fatty acid profile in the placebo group (Table 3). However, in the active treatment group levels of arachidonic, y-linolenic, and dihomogammalinolenic acids all increased significantly (towards normal values) by the end of the trial	
7	Arisaka [23]	8 months	PGE2 (pg/ml) → EPO: 63.9 ± 9.0, placebo: 59.5 ± 10.5 PGF2α (pg/ml) EPO: 189.2 ± 82.2,placebo: 173.6 ± 56.4	PGE2 (pg/ml) → EPO: 38.6 ± 7.2, placebo: 63.2 ± 11.2 PGF2α (pg/ml) EPO: 158.5 ± 42., placebo: 162.4 ± 61.5		EPO supplementation may be beneficial in diabetes by preventing various vascular complications of diabetes that may be related to altered EFA and PG metabolism. EFA studies in diabetes have shown that the levels and rates of formation of long-chain EFAs, such as DGLA, arachidonic acid, and eicosapentaenoic acid, are consistently low and levels of PGE2 and PGF2, are raised, and PGEl formation is impaired. EPO capsules significantly increased the DGLA level to the normal range. EPO also contains linoleic acid, but no significant changes in serum linoleic acid were demonstrated. Some of this extra linoleic acid would have been converted to GLA and DGLA. it is suspected that increased plasma PGEl production subsequent to increased serum DGLA (the precursor of PGEJ suppresses production of series 2 PGs, such as PGE2	
8	Bamford [24]					no differences in measurements of weight, triceps, skin-fold thickness, or blood pressure (systolic and diastolic, taken while the patient was seated) or in the ratings of appetite and stress taken at each of the three visits were found. only one change during the trial (statistically significant at the p < 0.03 level) were observed, an increase in plasma arachidonic acid during treatment with evening primrose oil. The change was observed in only a small group-children taking the low child's dose (n = 6)	Nausea and bloating occurred in five subjects taking EPO and in one case taking placebo. Hyperactivity was developed in three children taking placebo and only one child taking EPO
9	MANKU [25]	24 weeks				Treatment with Efamol produced highly significant elevations in 2o:3n-6 and 2o:4n-6. DGI.A became normal, but arachidonic acid remained well below normal even after 3 months' treatment. The C-22 n-6 fatty acids were unchanged as were all the n-3 EFAs	
10	SCHALIN-KARRILA	12 weeks				EPO had a statistically significant improvement in the overall severity and grade of inflammation and reduction in the surface area involved like dryness and itch. there was a significant but smaller improvement in the placebo group. EPO had no significant effect on the amount of TXB; released into the serum during blood clotting. Levels of plasma TXB; and PGE, were also not significantly altered by EPO After 6 weeks the level of 6-keto-PGF|, was significantly increased in the EPO group and decreased in the placebo group, when compared with pre-treatment levels, but after 12 weeks the levels were close to the pre-treatment values. EPO had no effects on oleic acid (18: in9), palmitic acid (16:0), 11,14,17- eicosatrienoic acid (20: 3n3)	No side-effects due to EPO were observed
11	WRIGHT [26]	12 weeks				Each final symptom score (after 12 weeks of drug or placebo) was subtracted from the initial symptom score for each patient, and these values were then compared for evening-primrose oil and placebo. In the low-dose groups (A and D) itch was the only symptom which responded better to evening-primrose oil than placebo. In the high-dose groups (B, C, and E) the patients’ assessments showed that the evening-primrose oil was significantly superior to the placebo with regard to itch, scaling, and general impression of severity	no side-effects were noted
12	Breth-jones [27]	16 weeks				At 16 weeks, the mean (SE; number of patients) improvements in Leicester scores were 8–48 (285; 33) for patients on epogam, 2–54 (289; 35) for patients on efamol marine, and 7–15 (2 88; 34) for those on placebo. On neither active regimen was mean improvement significantly different from placebo at 16 weeks (p = 074 for epogam, p = 0–26 for efamol marine). The only significant differences were in favour of placebo over efamol marine for responses of erythema and cracking	nausea, diarrhoea
13	Whitaker [28])	24 weeks After 16 weeks both groups were washed out for 8 weeks	IgE → EPO: 7 (255–1000 KU/I), Placebo: 7 (192–1000 KU/I)			At the end of active therapy, no statistical difference could be shown between 2 patient groups. at the end of 24 weeks, patients in EPO group, showed statistical difference in all paramteres while the placebo group showed constant statistical improvement of overall evaluation, redness, cracking and dryness	
14	EBDEN [29]	8 weeks				There was no effect of Efamol capsules on the control of asthma. There was no statistically significant difference in the mean morning or evening peak expiratory flow rates for the last fourteen days of each treatment period. There was no statistically significant difference for a similar period in the symptoms score or daily inhaled bronchodilator usage. Similarly, there was no statistically significant difference in the histamine challenge value	No side effects were noted from treatment with Efamol
15	Hederos [30]	16 weeks	IgE (kU/l) in eczema → EPO:322, Placebo: 422			Asthma: This study found no clinical effect on peak expiratory flow or overall asthma response, neither with placebo nor with Epogam/Eczema: Both groups of patients were substantially improved with respect to baseline but no significant differences between groups were observed. No significant differences between the treatment groups were shown in the use of steroid ointments, classified by the most potent class of steroid used, although there with time in the Epogam group. highly significant increases in the concentrations of DGLA and arachidonic acid (metabolites of GLA) in the Epogam group but no change in the placebo group. The routine haematological and biochemical analyses showed only one significant difference between treatments and that was for urate, but all serum urate concentrations remained within the normal range	Five patients receiving Epogam reported five adverse events and six patients in the placebo group reported seven adverse events. None of these were considered serious, and only one in each group was considered to be of possible effect
16	Blommers [31]	6 months				The decrease in days with pain was 12.3% for evening primrose oil and 13.8% for its control oil (P = 0.73); the decrease in days with pain was 15.5% for fish oil and 10.6% for its control oil (P = 0.28)	Gastric, Abdominal, Skin, Increase in body weight
17	Goyal [32]	four menstrual cycles				The mean baseline breast pain score (NDBP) was 22 (on a scale of 0–56), which was similar across treatment groups. Twenty-five percent of patients had moderate mastalgia at baseline, while 75% had severe mastalgia. By the end of cycle 4, mean scores had decreased to 13–15, a reduction from baseline of approximately 35% (Table 3). There were no statistically significant differences among the four treatment groups. During the open treatment phase, all groups showed a further statistically significant improvement. By the end of cycle 12, mean scores had decreased to 8–11. This constituted an overall reduction from baseline of approximately 58%. The profile of change during the open phase was comparable across the four treatment groups	Digestive system disorders, respiratory system disorders, body in general—general disorders, reproductive system disorders, musculoskeletal system disorders, skin disorders
18	Pye [33]	6 months	Overall, a grade I or II response was achieved in 165 (77%) of the 215 patients with cyclical mastalgia (danazol 70%, bromocriptine 47%, evening-primrose oil 45%, progestagens 15% Overall, a grade I or II response was achieved in 29 (44%) of the 66 patients with non-cyclical mastalgia (danazol 31%, bromocriptine 20%, evening-primrose oil 27%, progestagens 9%			Effective	
19	Qureshi [34]	three months to 1 year. (over a period of one year)				Results showed that out of 25 patients treated with OEP, 64% had a clinically significant response after three months of treatment, compared with 92% with topical NSAIDs	abdominal bloating, nausea, weight gain, headache, depression, giddiness, rash and bad taste
20	Nasri [35]	12 weeks	GSH (µmol/L) → EPO: 563.6 ± 138.4, Placebo: 470.5 ± 106 MDA(µmol/L) → EPO:2.1 ± 0.5, Placebo: 2.3 ± 0.8	GSH (µmol/L) → EPO: 626.3 ± 125.5, placebo: 469.8 ± 106.7 MDA(µmol/L) → EPO: 1.7 ± 0.4, placebo: 2.8 ± 1.6		significant increases in serum 25-hydroxyvitamin D (25(OH)D) (+ 10.7 ± 8.4 vs. − 0.5 ± 1.6 ng/mL, p < 0.001) and plasma total glutathione (GSH) (+ 62.7 ± 58.0 vs. − 0.7 ± 122.7 µmol/L, p = 0.01), while there were significant decreases in triglycerides (− 7.3 ± 23.8 vs. + 6.9 ± 26.3 mg/dL, p = 0.03), very low-density lipoprotein (VLDL) cholesterol levels (− 1.5 ± 4.7 vs. + 1.4 ± 5.3 mg/dL, p = 0.03), total/high-density lipoprotein cholesterol ratio (− 0.3 ± 0.4 vs. − 0.02 ± 0.4, p = 0.02), and malondialdehyde (MDA) concentration (− 0.4 ± 0.4 vs. + 0.5 ± 1.8 µmol/L, p = 0.008)	No side effects were reported following supplementation throughout the study
21	Farzaneh [36]	6 weeks				The percent of improvement in The frequency, severity and duration of hot flushes in the evening primrose group were 39, 42 and 19% compared to the placebo group, respectively 32, 32 and 18%. Although all three symptoms of hot flashes improved in the evening primrose arm, only the severity was significantly better in this arm compared to the placebo group (P < 0.05)	
22	Gateley [37]	4 months (4 months EPO/ 2 months placebo and then 2 months EPO)				In the patients with cyclical mastalgia, the proportions of the esters of the saturated fatty acids, palmitic and stearic acid, were significantly elevated. The proportions of the esters of the polyunsaturated EFAs, linoleic, dihomo-y linolenic (DGLA) and AA, were significantly reduced. The differences in the patients with non-cyclical mastalgia were in the same direction, but only the reduced proportion of AA was significant. Evening primrose oil produced a significant increase in the proportion of DGLA, the metabolite of GLA, after 2 and 4 months of treatment	
23	Gateley [37]	12 months				Treatment with evening primrose oil produced an immediate significant increase in the proportion of DGLA, which returned to the pretreatment proportion 4 months after completing treatment. There was a gradual increase in the proportion of arachidonic acid, which became significant after 12 months and was maintained 4 months after completing treatment. The proportion of the saturated fatty acid palmitic acid fell gradually, the difference becoming significant at 12 months. Treatment with placebo led to similar changes in arachidonic and palmitic acid to those seen in the group treated with evening primrose oil, but no change in DGLA during treatment	-
24	Gupta	12 weeks (4 weeks baseline washout period, treated for 6 weeks, followed by a 2 weeks.)	CRP → EPO:3.56 ± 1.64, placebo: 4.17 ± 2.95 MDA (µmol/L) → EPO:4.58 ± 2.08, Placebo: 4.41 ± 2.19 SOD(U/gHb) → EPO: 982.20 ± 191.28, placebo: 978.02 ± 236.61 GPX(U/gHb) → EPO: 74.19 ± 32.2, Placebo: 52.29 ± 29.91	CRP → EPO:3.28 ± 1.57, placebo: 4.50 ± 2.96 MDA (µmol/L) → EPO: 4.18 ± 1.95, placebo: 4.71 ± 2.12 SOD(U/gHb) → EPO: 953.97 ± 188.66, placebo: 1033.63 ± 244.83 GPX(U/gHb) → EPO: 66.47 ± 29.44, placebo: 60.10 ± 28.91		significant reductions in LDL cholesterol (LDL-C; -17.33% of baseline, P < 0.001) and total cholesterol (TC; -13.38% of baseline, P < 0.0001) values were observed during the experimental treatment period. producing product. This treatment also led to a decrease in the levels of C-reactive protein (CRP), malondialdehyde (MDA) and superoxide dismutase (SOD), which are indicators of oxidative stress	-
25	Ishikawa [38]	16 weeks	Apo A-1 (mg/dl) → EPO:132 ± 12, Placebo: 132 ± 16 Apo A-2 (mg/dl) → EPO: 38.1 ± 6.5, placebo: 38.1 ± 8.1 Apo B (mg/dl) → EPO: 142 ± 37, placebo: 159 ± 17 Apo C-2 (mg/dl) → EPO: 6.3 ± 2.4, placebo: 6.7 ± 2.3 Apo C-3 (mg/dl) → EPO:13.6 ± 5.7, placebo: 14.2 ± 5.3 Apo E (mg/dl) → EPO: 6.7 ± 1.5, placebo: 6.9 ± 2.2			a significant decrease in low density lipoprotein-cholesterol and plasma apolipoprotein B compared with the levels observed during safflower oil administration. Our results confirmed that EPO is effective in lowering low density lipoprotein in hypercholesterolemic patients	–
26	JENKINS [39]	12 months	-			After 12 months there was no significant difference in mean serum AlT between the groups nor in the number of patients with AlT levels within the normal range. One of the 10 patients in the treatment group showed histological improvement, but seven showed no change and two deteriorated. This contrasts with the patients receiving placebo, of whom four showed spontaneous histological improvement and five no change	No side effects were seen
27	Khoo [40]	6 months	-			There was no evidence of a difference between total PMS scores of the active and placebo groups, the mean difference being—0.026 with a standard error of 1.144 (test statistic from paired t-test = 0.02; 37 df; P = 0.982). Similarly, the two treatment groups did not significantly differ in their scores for psychological (mean [SE) = 0.447 [0.630)), fluid retention (0.211 [0.422)), breast (- 0.053 [0.410)), or menstrual symptoms (0.053 [0.258)). The test statistics are, respectively,—0.71, 0.50,—0.13, 0.20 (37 df; P = 0.482, 0.621, 0.898, 0.840)	Had no toxicity
28	Kokke [41]	6 months				improved symptoms and overall lens comfort in female patients with contact lens-related dry eye. Supplementation also caused a significant increase in tear production, as defined by tear meniscus height. It is reasonable to assume that the observed clinical improvement is primarily due to the documented anti-inflammatory effects of these EFAs	
29	Laivuori [42]	31–36 weeks	-keto-PGF,,, 2,3-dinor-6-keto-PGF,,)/ (TXB2, 2,3-dinor-TXB,)			Supplementation of the diet with primrose or fish oil caused no changes in the production of PGI2, or TXA2 metabolites. The dietary supplementation appeared to have no effect on blood pressure or on other clinical variables, such as proteinuria and oedema	
30	MOODLEY	2 weeks				No significant differences were found between the groups in respect to perinatal outcome, blood pressure lowering effect and haematological indices	-
31	Makrides [43]	6 weeks				Supplementation of infant formula with FO (0.36% total fatty acids as DHA) resulted in DHA levels being elevated above those of breast-fed infants at 16 and 30 weeks	
32	Manthorpe [44]	3 weeks				Efamol/Efavit significantly improves the Schirmer-I-test in patients suffering from primary Sj6gren's syndrome (]'he Schirmer-I-test improved significantly during Efamol treatment while the P-values for the other tests did not reach the 0.05 level.). It has been suggested that the effect of Efamol is due to an insufficient amount of unsaturated fatty acids in the different tissues	Sudden universal flushing which usually began in the face and throat, sensation of heat, increase in pulse frequency and fear
33	OLIWIECKI [45]	28 weeks (placebo all patients first 4 weeks)				LAS scores for active and placebo-treated groups were compared at each visit. No significant difference was seen in the scores for erythema or scaling, e no significant differences between the active and placebo groups in the scores for itch, redness, anxiety and depression, no significant difference in plaque thickness and transepidermal water loss between the active and placebo-treated groups	-
34	Theander	6 months				No statistically significant improvement was found in fatigue assessed by Visual Analogue Scale (VAS) or in the time needed for sleeping/resting during a 24-h period. No differences were found between the treatment and placebo group. The same applies to the secondary endpoints: no differences in VAS for eye and mouth dryness or pain, no significant changes in Schirmer-1-test, van Bijsterveld score, unstimulated whole sialometry (UWS), or use of artificial tears or analgesics	mild gastrointestinal some patients complained about weight gain
35	Ka ´zmierska [46]	9 months				Compared to isotretinoin treatment, isotretinoin treatment combined with EPO had a positive effect on TCH concentrations (mean: 198 vs. 161, p < 0.001), LDL (95.9 vs. 60.2, p < 0.001), HDL (51.0 vs. 48.0, p < 0.001), TG (114 vs. 95.0, p < 0.001), ALT (24.0 vs. 22.0, p < 0.001), and AST (28.0 vs. 22.0, p < 0.001), but had no effect on the energy and ingredient content of the diets (p > 0.05) after treatment	No side effects were reported
36	Oxholm [47]	16 weeks				the results from Schirmer-I test, break-up time and van Bijsterveld score, improved significantly during Efamol treatment when compared with Efamol start-values. The GLA metabolite and prostaglandin-El (PGE,) precursor dihomogammalinolenic acid (20: 3116, DGLA) increased both in plasma and in erythrocytes) during treatment with Efamol. No correlations between objective ocular and oral status and DGLA values in plasma or erythrocytes were found	transient nausea and softening of stools: 3 patients
37	Iran/Soheila Rezapour-Firouzi a,b, ∗ , Seyed Rafie Arefhosseini	6 months	IL-4 (pg/ml) → A: 0.56 ± 0.20, B: 0.50 ± 0.50, C: 0.81 ± 0.12 IFN-γ → A: 0.56 ± 0.04, B: 0.22 ± 0.06, C: 0.35 ± 0.23, IL-17 → A: 0.51 ± 0.09, B: 0.26 ± 0.11, C: 0.51 ± 0.03	IL-4 (pg/ml) → A: 0.70 ± 0.17, B: 0.41 ± 0.14, C: 0.96 ± 0.11 IFN-γ → A: 0.24 ± 0.04, B: 0.39 ± 0.06, C: 0.30 ± 0.14, IL-17 → A: O.39 ± 0.04, B: 0.41 ± 0.20, C: 0.45 ± 0.15		combination of HSO and EPO as a dietary supplement in a daily dose of 18—21 g/day over a period of 6 months showed immune-modulating effects in our study with RRMS patients resulting in significant improvements of the EDSS score and the relapse rate compared to a control group receiving 18—21 g olive oil per day. Small changes in the levels of the cytokines were observed in all groups and were rather consistent with the clinical outcomes: IL-4 increased significantly in group A and C, IFN-γ decreased significantly in group A and increased in group B. The Mizadj score increased in both active treatment groups significantly. Further research must show the properties of this score and its the correlation with the clinical data	-
38	Rezapour-Firouzi [48]	6 months	-			There was no significant difference in the study parameters at baseline. Serum levels of liver enzymes (GGT, AST, and ALT) were serially monitored. Intervention was associated with liver function alteration in three groups. Significance decreased in EDSS score and the levels of liver enzymes were found in groups A and C, whereas elevated serum liver enzymes and EDSS score were observed in group B after the intervention	-
39	Rezapour-Firouzi [49]	6 months	-			After 6 months, significant improvements in EDSS and functional score were found in the groups A and C while EDSS and pyramidal score showed significant increase in group B. Alteration of biochemical parameters showed improvement in groups A and C whereas there was worsening condition for group B after the intervention. (the observed reduction of D6D was a consequence of the well- described effects of this type of intervention, and that an increase in PUFAs and reduction in expression of sPLA2 key enzymes caused a decrease in mean EDSS. Surprisingly, altering PUFAs rate causes a decrease in sPLA2 expression, in particular, in the co-supplemented oils and Hot-natured diet group)	-
40	Rezapour-Firouzi [50]	6 months	IL-4 (pg/ml) → A: 0.58 ± 0.50, B: 0.50 ± 0.50, C: 0.81 ± 0.87 IFN-γ → A: 0.26 ± 0.04, B: 0.22 ± 0.06, C: 0.35 ± 0.23	IL-4 (pg/ml) → A: 0.69 ± 0.69, B: 0.41 ± 0.14, C: 0.95 ± 0.91 IFN-γ → A: 0.24 ± 0.04, B: 0.24 ± 0.06, C: 0.31 ± 0.14		There was no significant difference in the study parameters at baseline. After 6 months, EDSS, Immunological parameters and the erythrocyte cell membrane with regard to specific fatty acids showed improvement in the group A and C, whereas there was worsening condition for the group B after the intervention. We concluded that Hot-nature dietary intervention with co-supplemented hemp seed and EPOs caused an increase PUFAs in MS patients and improvement in the erythrocyte membrane fatty acids composition. This could be an indication of restored plasma stores, and a reflection of disease severity reduction	-
41	Vaddadi [51]	8 months (32 weeks)				The efficacy/anti-dyskinetic effect of EFA supplementation was marginally significant but not clinically significant. However, active treatment produced highly significant improvements in total psychopathology scores and schizophrenia subscale scores and significant improvements in memory	No side effects
42	VEALE [52]	12 months	ESR, CRP, TXA2			all measures of skin disease activity including severity, percentage of affected body and itching remained unchanged by Efamol Marine. NSAID requirement remained the same between both treatment groups. In addition, no changes were shown in arthritis activity as measured by duration of morning stiffness, Richie joint index, number of active joints, ESR and CRP. However, an increase in serum TXB2 was observed in the active group in the placebo phase. Furthermore, there was a decrease in leukotriene B4 production during the active phase followed by a significant increase during the placebo phase, suggesting some in vitro documented anti-inflammatory effects	-

**Table 3:** Characteristics of included clinical trial studies in the systematic review of EPO effectiveness (topically administered) on inflammatory diseases

**No.**	**Author**	**Type and duration of disease**	**Number of participants**		**Female/Male ratio**		**Age**		**Type of intervention and dosage**		**Duration of intervention**	**Outcome**	**side effect**
			Intervention	control	Intervention	control	Intervention	control	Intervention	control			
**1**	Gehringa [51]	Atopic dermatitis	10	10	4/6	8/2	average age of 25.1 years (range: 19 to 42 years; median of 24 years)	mean age of 22.9 years (range: 18 to 42 years; median of 23.5 years)	entire flexor side, morning and evening (emulsion 1)	emulsion 2	5 weeks (4 weeks treatment)	However, the present study is characterized by the fact that a statistically significant stabilizing effect on barrier performance was observed with the EPO fraction relative to the vehicle, recorded as a decrease in TEWL. The peak effect was not evident for 5 weeks, including a 1-week treatment-free period. Therefore, this study proves that the onset of a long-term interaction with the lipids of the epidermal barrier is slow beyond the physical properties of EPO. Unlike water-in-oil emulsions, amphiphilic oil-in-water emulsions are unsuitable vehicles for EPO because no effect was demonstrated above and beyond that vehicle alone	-
**2**	OLIWIF.CKl [52]	Epidermal atrophy/ healthy volunteers	12	12			21–54 years		two tubes of cream, one to be applied to the right forearm and one to the left (twice daily) cream B: betamethasone valerate + EPO and Cream A: betamethasone valerate	two tubes of cream, one to be applied to the right forearm and one to the left (twice daily) cream c: arachis oil) and Cream A: betamethasone valerate	3 weeks	when creams A and H (betamethason EPO) were compared, no significant difference was observed in the thickness of the epidermis (P > 0–2)	-
**3**	Ratz-Łyko1 [53]	healthy volunteers	15				18–55 years		Twice a day on skin area Oenothera biennis and borago officinalis	Twice a day on skin area Nigella sativa seedcake extracts and placebo	6 weeks	Reducing skin irritation and improving skin hydration and epidermal barrier function	-

### Assessment of risk of bias

Three reviewers assessed the risk of bias in the included studies with the standard summarized tool in the Cochrane Handbook [52]. This tool assesses six domains related to the risk of bias (random sequence generation, allocation concealment, blinding of participants and personnel, blinding of outcome assessment, incomplete outcome data,) and categorized studies by low risk, unclear risk or high risk of bias in each domain. For the random sequence generation domain, if the generation of a random sequence was non-randomly performed, the risk of selection bias was considered to be high. If a random component was described in the process of sequence generation, the selection bias would be considered as low risk; and if it was not explained in sufficient details, the selection bias was considered to be unclear. For the allocation concealment domain, if the participants or investigators could possibly foresee assignments, the risk of bias was considered to be high while if they could not foresee assignment, it was considered to be low. If the method of concealment was not described in sufficient detail, unclear risk of bias would be selected. For the blinding domain, it would be considered to be high if incomplete blinding or no blinding was done and the participants and personnel were aware of the interventions during the study, and low if blinding of investigators and participants was ensured. In case of insufficient information or not addressing this outcome, it was considered unclear. If there was no missing outcome data or in case of the existence of missing data, they were imputed using appropriate methods, this domain was considered as low; otherwise it was considered as high. Insufficient reporting of attrition/exclusions made this domain to be selected as unclear. In the Selective reporting domain, if not all of the pre-specified primary outcomes based on the study protocol was reported, high risk was selected. If the study protocol was available and all the outcomes was reported, it was considered as low risk. Moreover, if the study protocol was not available but it was clear that the published reports included all expected outcomes, it was also considered as low risk. In case of insufficient information to permit judgement it was unclear. ​ Disagreements in risk of bias assessment were resolved through consensus. Quality assessment of included studies was conducted with the risk of bias table in RevMan 5.3 for RCTs.

### Grading of the evidence

The JBI Grades of Recommendation framework provides a structured and transparent method for evaluating the quality of evidence and making recommendations based on that evidence. The JBI Grades of Recommendation framework consists of the following levels: Grade A: There is strong evidence to support the recommendation. This level is assigned when there is consistent high-quality evidence from multiple studies. Grade B: There is moderate evidence to support the recommendation. This level is assigned when there is limited or inconsistent evidence from multiple studies or strong evidence from a single study. Grade C: There is weak evidence to support the recommendation. This level is assigned when there is limited evidence from a single study or expert opinion. Grade D: There is insufficient evidence to support or refute the recommendation. This level is assigned when there is a lack of available evidence [53] (Appendix).

## Results

Based on the search strategy, 424 articles were obtained, and after removing duplicate articles, 103 studies remained. A complete review of the remaining articles led to the removal of 46 articles that did not have enough information or were irrelevant. Out of the remaining 57 articles, 12 articles were removed in the title and abstract section due to not mentioning the theoretical content or being unrelated. Finally, 44 articles was entered this systematic review and under the next subsection, which is categorized by the conditions, they will be further discussed.

**Fig. 1:**
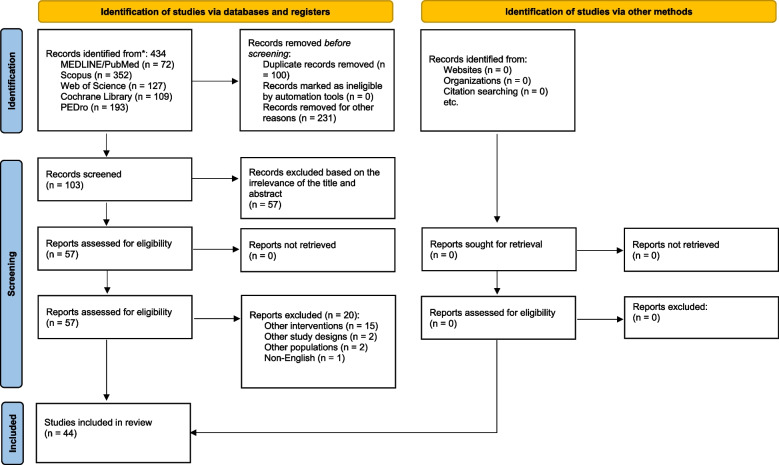
PRISMA flowchart of the study

The PRISMA flow chart is shown in Fig. 1.

### Risk of bias within studies

**Fig. 2:**
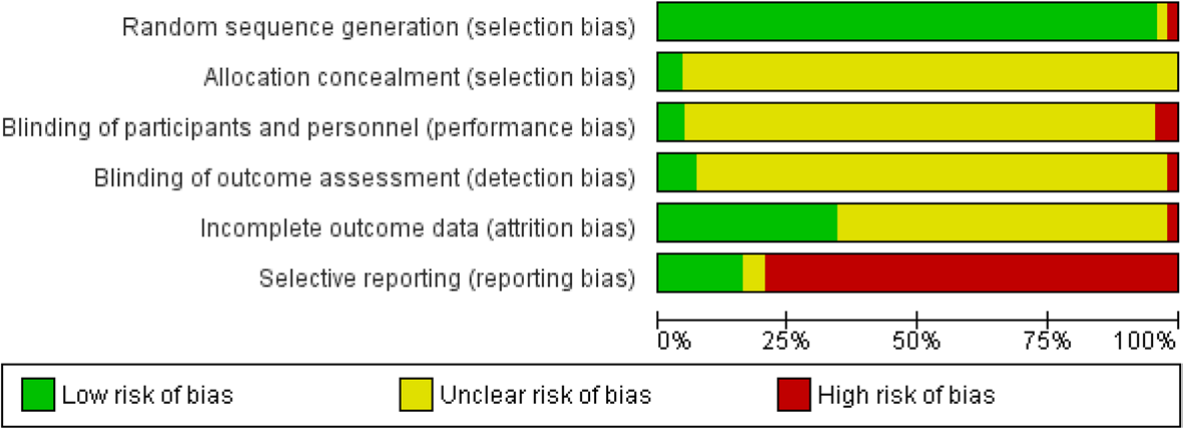
Risk of bias in randomized controlled trials using Cochrane risk of bias tool

**Fig. 3:**
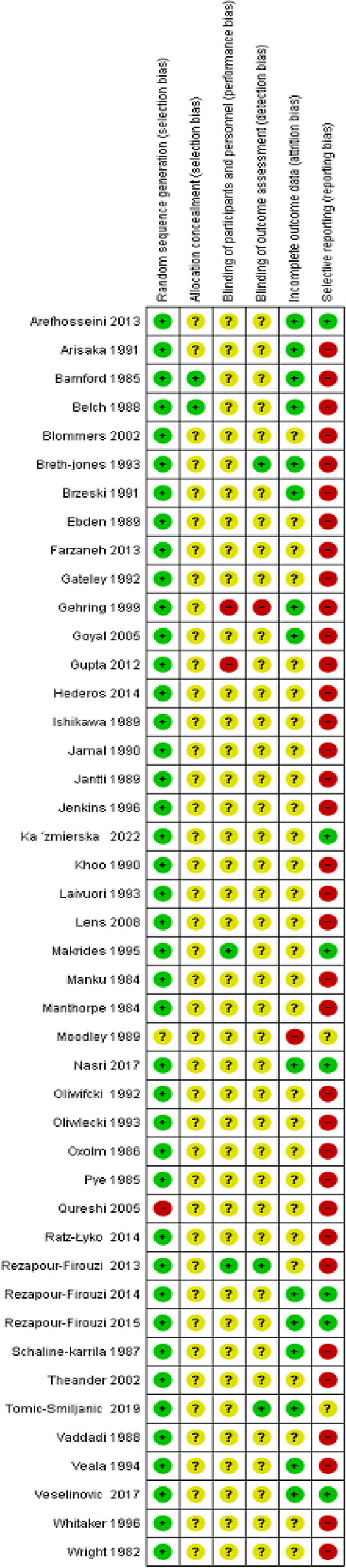
Summary of risk of bias in selected studies (Cochrane Risk of Bias Tool for controlled trials)

The quality of the studies that are reported in our article were determined using the Cochrane Risk of Bias (Figs. 2 and 3). Accordingly, forty-two studies (95%) with low risk, one study (2.27%) with high risk, and one study (2.27%) with the unclear risk of bias for random sequence generation**.** For allocation concealment, forty-two studies (95.45%) had unclear bias and two studies (4.5%) had low bias.

In the case of blinding of the participant: forty studies (90.90%) had unclear bias, and two study (4.54%) had low risk and 2 studies (4.54%) had high bias. Blinding of outcome assessment was high risk in one study (2.27%), and low risk in three studies (6.81%) and unclear risk in forty (90.9%). In attrition bias, 63.63% of the studies were unclear risk, 34.09% were low risk, and 2.27% of the studies were high risk.

Meant for reporting bias, thirty-one studies (79.54%) had high risk, eleven studies (15.90%) had low risk and two studies (4.5%) had unclear risk.

### Results of the individual studies

#### RA

RA damages cartilage and bone by involving the joints, which has an incidence of about 0.5–1% and is known as one of the most currently known autoimmune diseases. The pain and swelling of the joints are caused by the initiation of pathologic alterations, via the entrance of B cells, macrophages and other factors to the synovial liquid [15, 54]. These alterations cause the overproduction of inflammatory factors including tumor necrosis factor (TNF), IL-1, PGE2, and cytokines bringing about chronic inflammation. GLA, the most abundant constituent of EPO, has anti-inflammatory effects through being converted to di-homo-γ-linoleic acid (DGLA) that stops the switching of AA to LT and the production of PGE-1, which plays important anti-inflammatory roles in RA [15, 55]. Five clinical trials with a study duration range of 3–12 months evaluated the effects of EPO on the improvement of RA. The patients received EPO in the form of oral capsules every day [15, 54–57]. Three of them asserted that EPO was effective and showed significant improvements in symptoms of the disease, one of them stated that EPO had mild effects and others reported no significant effects [15, 55–57]. In one of the studies, mild gastric discomfort (diarrhea, nausea, and pain) was reported as the side effect of EPO [54]. Also, a letter claimed that a significant effect did not appear with consumption of EPO [58]. In summary, it seems that EPO might be effective in terms of RA treatment.

#### Diabetes mellitus (DM)

DM has an increasing prevalence recently, and could affect different organs such as the kidney, brain, heart, and others, in chronic stages. It is diagnosed by different factors and is classified into 7 types. DM should be controlled by pharmacotherapy and lifestyle enhancement to reduce the risk of cardiovascular disease and the relative mortality [59]. Some experiments claimed that abnormalities in red cell n-6 polyunsaturated EFAs and serum platelets could be increased in animals and humans with DM. The reduction of insulin levels blocks 6-desaturation and impairs the conversion of LA to GLA. This phenomenon has a key role in membrane structure and 6-desaturated metabolites, which are known as precursors of PGs and eicosanoids. All of these can lead to cardiovascular complications in the course of the DM disease. Consequently, it was suggested that high doses of LA and GLA could be effective in neuropathy improvement and cardiovascular disorder risk reduction [17]. Two clinical trial studies reported the impact of EPO on DM. One of them prescribed EPO and placebo capsules to 22 patients for 6 months, twice daily where significant positive clinical and neurophysiological effects and polyneuropathy improvement were observed [17]. Also, in the other study, with an intervention duration of 8 months, EPO capsules had beneficial effects on DM and vascular complications [17]. In conclusion, a high dose of oral EPO was effective and improved the disease condition in DM.

#### Atopic eczema (AE)

AE is one of the most periodical inflammatory skin diseases showed a growing prevalence which could reduce the quality of life of the patients with this disease[60, 61]. Typically, AE is known as an immune system-related disease that mainly occurs in patients with a family or/and personal history of atopy and the lesions with different symptoms are its symptoms [62]. Epidermal barrier deficiency is one of the most important reasons for the disease pathology and a route to its treatment. This can induce an increase in the permeability of the stratum corneum which causes more irritation by noxious substances and additive immunologic skin activation by increasing allergen entrance [49]. The increasing level of LA and decreasing level of its derivatives confirmed that δ-6-desaturation is impaired [18]. Indeed, inhibiting the cis-LA conversion to GLA induces the reduction of DGLA, the precursor of PGE1 that has an important role in the normal activity of T-lymphocytes (impaired T-lymphocyte function is clear in AE), and AA the precursor of PGE2 [18, 63]. AA and DGLA are appropriate and essential for the structure and normal activity of the skin [63] Even though topical steroids seem to be more effective than EFA supplements in AE, the final product of δ-6-desaturation could be of value in relief of AE [18, 63]. From 11 clinical trials that have been carried out in this case, 9 of them showed improvement and 3 of them claimed that EPO was not effective in the treatment process in AE (one of these studies used EPO as a topical emulsion despite other studies that used the oral capsules) [18, 19, 21, 49, 63, 64]. Consequently, it seems that EPO can be effective in AE through oral administration.

#### Chronic hands dermatitis

As far as we know, chronic hand dermatitis with a prevalence of 2–44%, has multifactorial pathologic reasons. Increasing the skin permeability irritates with materials that do not lead to any reaction in ordinary conditions. The first line of treatment is recognition of the cause of the disease (possible allergens or irritating substances) and preventing their associations. Topical or systemic steroids and sometimes antibiotics are used as the drug treatments of the choice. Despite these pharmaceutical treatments, EPO supplementation could be effective via the role of GLA in increasing the water permeability in the epidermal barrier. In the only clinical trial that had been conducted on the chronic hand dermatitis cases having a duration of 24 weeks, 39 patients consumed oral EPO and the results did not show effectiveness [22].

#### Tardive dyskinesia (TD)

Tardive dyskinesia, one of the most serious complications in the patients with schizophrenia, has various prevalence related to age (from 4–5% in younger to about 24% in older people). Different factors such as age, smoking, psychiatric disorders, and others contribute to the risk of TD [17]. EFAs have a non-negligible role in the structure and normal function of the membranes and also approximately 15–20% of the dry weight of the brain that is formed by them. EFAs derivatives (PGE1, PGE2) antagonize the dopamine function by cyclic adenosine -3’,5’- monophosphate, thereupon, EPO could block the hyperactivity of dopamine causing TD by conversion to DGLA and AA (precursors of PGE1 and PGE2). In one study, 38 patients used EPO capsules for 32 weeks and marginal improvements were obvious, however, it was reported that the mentioned improvements were not clinically that important [47].

#### Psoriatic arthritis

Psoriatic arthritis, one of the common skin disorders, with a prevalence of 1–2%, impacts distal sites of hands and feet and has asymmetric joint distribution [48, 65]. The skin lesions, mainly appear in the hairline, natal cleft, ears, umbilical area, and groin. Also, nail lesions can lead to the true diagnosis [65]. Impaired AA metabolism increases the AA and LTB4 levels in patients’ lesions. Thus, EPO might improve the lesions. In a clinical trial with 38 participants and a duration of 12 months, patients consumed EPO capsules once daily, but all the factors measurements remained unchanged [48]. In conclusion, it seems that despite the effective mechanism of EPO on psoriatic arthritis, it was not profitable for patients in this study.

#### Asthma

Asthma is categorized as one of the most common chronic inflammatory diseases with an increasing prevalence and morbidity that is activated by many different inflammatory cells in the airways. PGE1 as the final product of GLA conversion, is a bronchodilator and more stable than PGE2. Thus, EPO could create positive effects in this type of inflammatory disease [23]. Two clinical trial studies with a duration of 8–16 weeks, have tested oral EPO capsules on asthmatic patients in both no significant positive results were obtained [23]. We might conclude that EPO seems not to be effective in asthma. (As a result, despite the hypothesis that the drug is effective, no acceptable results were reported from these studies.

#### Polycystic ovary syndrome (PCOS)

Polycystic ovary syndrome, with an incidence of 5–15%, is generally caused through an impaired function of endocrine system (mostly ovarian hyper androgenism) in aged women, bringing about varied symptoms such as obesity, hirsutism, and acne [66]. Assorted complications such as, high levels of low-density lipoproteins (LDL), low concentration of high-density lipoproteins (HDL), and high body mass index (BMI), accompany PCOS and increase the risk of type 2 diabetes mellitus (T2DM) and coronary heart disease (CVD) in patients suffering from PCOS. Supplements containing Vitamin D could recuperate lipoproteins concentrations and vitamin D levels in PCOS patients. More to the point, EPO has direct and indirect effects on synthesis of immune cells and eicosanoid. It has been reported that co-supplementation of vitamin D and EPO, in the form of oral capsules, on 60 patients and for 12 weeks, exhibited significant effects on the assessed parameters, claiming that EPO would be effective in PCOS [29].

#### Cystic fibrosis (CF)

Cystic fibrosis, a generalized endocrinopathy (affected exocrine glands), is recognized by the protein and fat malabsorption which can be caused via inhibition of pancreatic enzyme secretion leading the disease process into lung infection. Correspondingly, growth failure and steatorrhea are the diagnostic signs. Nutritional repletion, airway infection treatment, airway obstruction relief, suppression of inflammation, and lung transplantation (the main cause of morbidity and mortality in CF) are the choices of treatment [67]. Given that EFA deficiency is widely reported in patients, predominantly due to the impaired function of pancreatic beta cells, EPO supplementation might be effective in CF. Nonetheless, in a clinical trial, with 16 participants and 12 months’ duration of intervention, patients took oral EPO supplementations daily and no improvements were observed in the symptoms of CF. In view of that we might reject the theory of EPO effectiveness in CF [68].

#### Menopausal hot flashes

Peripheral vasodilation, increases blood skin flow coming with flushing, sweating (particularly in the chest, face, and neck), and chills. It has been anticipated that the duration of menopausal symptoms is about 4–20 years, and the prevalence is lower in Japanese and Chinese women. Despite the fact that every course of hot flashes usually takes a little time (1–5 min and sometimes 1 h), reduction in quality of life is the momentous issue. Therefore, different treatment options have been introduced such as behavioral treatment and drug therapies; clonidine, serotonergic agents, and etc., that are mainly divided into two categories, hormonal and non-hormonal medicines [69]. EPO consumption is one of the non-hormonal ways of treatment in menopausal hot flashes. The mechanism of action for EPO is not clear yet in this case, but some references suggest it for alleviation of the symptoms. In a clinical trial, 56 aged women consumed t EPO capsules, every day for 6 weeks and significant improvement appeared in the symptoms deducing its effectiveness in menopausal hot flashes [30].

#### Hepatitis B

Hepatitis B is a chronic infection caused by the hepatitis B virus, with variable incidence, has effective and safe vaccine prevention. It is identified by hepatocellular damage, advanced fibrosis, and infiltration of inflammatory cells [70]. As mentioned in the previous parts, EFAs have essential role in membrane functions and their supplementation would improve histological and biochemical parameters in liver disease. In a 12-month trial study, researchers evaluated oral EPO capsules effectiveness on 10 patients with hepatitis B and the achieved final result was not satisfactory [71]. In conclusion, it appears that the offered treatment could not be acceptable for patients with hepatitis B.

#### Premenstrual syndrome (PMS)

Premenstrual syndrome are a set of symptoms including fatigue, breast tenderness, abdominal pain, depression, and etc., affect a large number of women before menses reducing the quality of life to a great extend [72]. Despite the different treatment choices like hormones, anti-prostaglandins, vitamins, EPO supplementation is suggested owing to the PGE1 positive effect on abnormal sensitivity to the prolactin level in blood circulation in patients with PMS. A clinical trial study was designed for 38 patients and oral EPO capsules were tested for 6 months. The findings of that study revealed that there were no advantages in EPO consumption in comparison with the placebo [34]. It looks like EPO might not be effective in reducing PMS symptoms.

#### Contact lens-associated dry eyes (CLADE)

A common complaint among contact lens users, which could be related to eye environment or the design and material of the lens. Variable symptoms such as blurred vision, eye fatigue, and prolonged dryness are experienced by patients more in the evening or night [73]. Despite the beneficial treatments including anti-inflammatory and immunomodulatory drugs like cyclosporine, it has been reported that the final products of EPO metabolisms have anti-inflammatory effects on CLADE. In a controlled clinical trial study 52 participants with contact lens-associated dry eyes consumed oral capsules of EPO for 6 months. The outcome of the study showed no significant differences between the placebo and intervention groups [35]. Accordingly it is suggested that EPO is not effective in this case.

#### Acne vulgaris

Overproduction of sebum affects the pilosebaceous unit and causes this chronic inflammatory disorder. Inflammatory (papules and pustules) and non-inflammatory lesions are the disease manifestations that are more aggregated in the neck, shoulders, face, upper chest, and back. Depending on acne severity, topical benzoyl peroxide, antibiotics and retinoids, and oral contraception or antibiotics, in combination or individually, could be effective [74]. Isotretinoin consumption causes abnormalities in lipid profiles and LA reduction is noticeable. EPO consumption showed improvement in lipid profiles. A clinical trial study was designed for 9 months, and biochemical parameters were assessed during EPO and placebo capsules consumption. In the end, improvement in lipid profile was clear in comparison with isotretinoin [42]. After all, it is advocated that EPO supplementation could improve the disease condition in acne vulgaris.

#### Mastalgia

A common breast pain in women that mostly occurs before menstruation which is normal and physiological or is severe and nodularity which could reduce the quality of life and relationship with partner and the children and about 70% of women complain about it [28]. Treatment choices includes danazol with the best response (70%) and low side effects (22%) but the most expensive price, bromocriptine with 45–47% of positive response, more side effects, and lower price in comparison to danazol, are available [42]. Besides, decreasing the fat in dietary and stopping hormone replacement therapy and oral contraceptive consumption are the other effective supportive treatments [40]. Despite all these therapy methods, EPO with uncommon adverse effects (2%), is the first choice for most patients because of the lower relapse rate and side effects of hormonal therapy [27]). On the other side, EPO affects the prostaglandin metabolism and can improve the mentioned complaint [25]. Also, GLA deficiency is well-defined in mastalgia and since EPO is a rich source of GLA, it could be effective in the remedy for mastalgia [26]. In 5 clinical trials, patients used oral EPO capsules for different durations and 4 of them claimed that significant improvement were obtained as a result [25, 26, 28, 31]. Also, a letter prescribed that oral EPO in a study with 135 patients had no significant effects on patients with mastalgia [75]. Taken as a whole, we might claim that EPO supplementation might be effective in treatment of mastalgia.

#### Hypercholesterolemia

Impaired lipoprotein levels in plasma, increases the risk of cardiovascular and coronary heart disease (CHD). Some other risk factors such as diabetes, smoking, aging could play an important role in HDL reduction and LDL increment [75]. Even though EPO metabolites can reduce cholesterol and LDL levels, n-6 metabolites (considerably DGLA and AA) deficiency is another risk factor for CHD and low levels of LA, explain it [75]. Two controlled clinical trials studies were designed to assess the oral EPO consumption effect on lipoprotein level improvement and the results of them showed a reduction in LDL level [32, 75]. Consequently, EPO seems to be effective in down regulation of hypercholesterolemia.

#### Breast cyst

A common benign breast disorder, with an incidence of 70–90%, can be symptomatic or asymptomatic, small (microcyst) or large (macrocyst), single or multiple. Aspiration is one of the ways of treatment with periodic follow-up [32]. A deficiency of LA metabolites caused by an abnormality in the fatty acid profile is clear in patients with macrocysts. So, EPO can be profitable in disease amelioration. In a study, daily usage of EPO and placebo capsules were evaluated on patients for 12 months and no differences were found between the 2 groups. In view of that, we might declare that EPO might not be effective in treatment of breast cyst [31].

#### Pre-eclampsia

Proteinuria and high blood pressure that occur in 3–5% pregnant women, is the main cause of mortality in patients. Variable risk factors including hypertension, previous pre-eclampsia, diabetes, autoimmune disorders, and chronic kidney disorders influence the disease affliction. Owing to the fact that the treatment cycle delivers different complications for the mother and fetus, prevention is vital during pregnancy [76]. Reduction of prostacyclin (PGI2) and increment of thromboxane A2 (TXA2) is obvious in preeclamptic patients and AA could balance their levels [36]. Also, low level of PGs (prostaglandins decrease vascular sensitivity) in patients is suggestive of EPO supplementation [37]. In two studies, oral EPO capsules were evaluated on the preeclamptic patients and at the end of the intervention, no significant improvement was observed [36, 37]. It seems that the theory of EPO consumption as a way for pre-eclampsia treatment is deniable.

#### Multiple sclerosis (MS)

Evidently, MS is known as a common neurological disorder with an increasing prevalence that is multifactorial and many genes with different environmental factors such as smoking, obesity, and others, affect the affliction. Symptomatic pharmacotherapy to treat neurological dysfunction and MS-allocated remedies are available [77]. Impairment in the Th1 (the interferon-gamma (IFN-γ) producer)/Th2 (interleukin IL-4 producer) balance is one of the etiological risk factors. On the other side, IL-17, the product of Th17, has a key role in MS pathogenesis, and the derived cytokines of Th1 and Th2, suppress the Th17 development [61]. As it is stated in Traditional Iranian Medicine (TIM) warm temperament can create Th2-like immune responses, consequently, warm temperamented supplementation might be beneficial in autoimmune diseases that tend to Th1 immune responses (like MS). It has been reported that ω3-polyunsaturated fatty acids (ω3- PUFAs) can reduce IFN-γ generation in MS patients [45]. Since the current treatments (like; IFN-β1a, and IFN-β1b) are expensive in comparison with their effectiveness and cause side effects, natural supplementation like EPO possibly would be advantageous [46]. In a study, daily consumption of oral EPO and placebo was tested on 65 patients, and different factors were assessed after 6 months of drug administration. At the end of the study, all the assessed factors showed meaningful improvements [44–46, 61]. In a word, it seems that EPO can be helpful in MS.

#### Primary Sjogren's syndrome (SS)

Primary SS, is a common inflammatory disorder, with an incidence of about 0.04–4.8%, affecting the connective tissues with endocrine glands involvement [41, 78]. The regular symptoms are ocular and oral dryness and fatigue which is defined as mental or physical exhaustion and leads the disease process to the shrinked quality of life [41]. Reduced PGE1 levels and major products of LA conversion in erythrocytes and the important roles of EFAs in cell membranes, justify the EPO efficiency in this disease [43, 78]. Three clinical trial studies assessed the effectiveness of daily usage of oral EPO capsules, nonetheless, the succeeded findings were not satisfactory, since just in one study EPO was effective [41, 43, 78]. At long last, we might assume EPO could not be of value in primary SS.

#### Psoriasis

A chronic inflammatory skin disease, with environmental and genetic etiology factors, is divided into different types. Supposedly, stress, direct skin trauma, bacterial or viral infection, and other factors, are the affliction risk factors. Plaques, papules, and patchy lesions that sometimes are painful, manifest in different sizes on arms, legs, nails, and other parts of the body. Treatment should be individualized depending on the patient’s condition and topical corticosteroids, tazarotene, vitamin D analogs, and others, are the medicinal treatments of the choices [79]. Abnormality in EFAs levels and reduction in proinflammatory eicosanoids production and considering that the EPO derivatives prostaglandins have less inflammatory effects, EPO supplementation might be considered rewarding in psoriasis. In the study that was designed for 28 weeks, oral EPO capsules and a placebo were consumed by 37 psoriatic patients and no evident effect was observed [40]. In conclusion, despite the effectiveness of EPO according to the mentioned hypothesis, in this study it was not effective in psoriasis.

#### Healthy volunteers

The effect of EFAs on epidermal atrophy, seed cake on the skin, and plasma fatty acid levels in humans, was evaluated on healthy volunteers.

- The effect of EFAs on epidermal atrophy: EPO and placebo in the form of topical formulation were tested on 24 healthy volunteers for 3 weeks and the result claimed that EPO was not effective on atrophy and no significant effect was observed [50].
- The effect of seed cake on skin: a topical formulation of EPO and placebo were tested on 15 healthy volunteers for 6 weeks and significant positive effects were seen. EPO reduced skin irritation and improved the barrier function by hydrating the skin [69].
- Plasma fatty acid levels in humans: this study was designed for 10 days and, oral EPO or placebo were taken by 76 healthy volunteers. The result of the study showed that EPO was harmful to inflammation due to the increasing levels of AA [80].

### The strengths of the evidence

According to the JBI guidelines (Appendix), based on the available evidence, the recommendation for using EPO for inflammation control is weak. This conclusion is supported by the fact that the included studies are rather inconsistent and heterogeneous and there is lack of high quality studies. Larger sample sizes and narrower confidence intervals provide more precise estimates, increasing the certainty of evidence.

## Discussion

As far as we know, *O. biennis* known as evening primrose is a medicinal plant from the Onagraceae family. The *O. biennis* seeds oil mainly contains active biological components including fatty acids, polyphenols, aliphatic alcohol, and sterols. It has been reported that the main components of EPO were LA (70–74%) and GLA (8–10%) demonstrate anti-inflammatory activity via different mechanisms. DGLA, the product of GLA, follows the two below mechanisms in exhibiting anti-inflammatory effects:

1. DGLA oxidation via lipoxygenase (15-LOX) and producing 5-hydroxyeicosatrienoic acid (15-HETrE)
2. DGLA conversion to PGE1 via cyclooxygenase (COX)

Both of the final products in the above mentioned pathways possess anti-inflammatory activities. Likewise, 15-HETrE inhibit the AA conversion to LTA4 by blocking the 5-LOX enzyme and GLA reducing the following inflammation mediators’ levels: IL-1β, IL-6, and TNF-α [81].

In this systematic review, based on our search strategy, from the initial 424 articles we selected 44 clinical trial studies in which they evaluated EPO effect on different types of inflammatory disorders. 41 studies used EPO in the form of oral capsules and 3 studies tested the EPO in topical form. Although, about 28 clinical trial studies showed significant positive effects, nearly 10 types of diseases showed the opposite result. The most positive results were obtained in oral administration of EPO in AE, mastalgia and RA. As far as we know, depending on AE severity, variable regimens are recommended for treatment. Consumption of supplementations containing fatty acids (fish oil or EPO) besides, skin hydration, avoidance of skin stimulants and anti-inflammatory drugs including topical glucocorticosteroids and calcineurin inhibitors are the most advised treatments in AE. On the other hand, mastalgia is characterized with unclear etiology, decreasing the quality life of women specially before menstrual cycle. Unbalanced ratio of unsaturated (deficiency) and saturated fatty acids in patients, gives us a clue that PUFA supplementation could improve symptoms of disease, highlighting the role of EPO in treatment of mastalgia [82]. Likewise, in the case of RA, combination of pharmacological and nonpharmacologic treatment base on the stage of the RA, reduce the symptoms and increase the quality of life. The supplementation of PUFAs could relief inflammation and based on the mechanism which has been mentioned in results, EPO seems to be much effective in RA. More to the point, it was publicized that EPO was also effective in a few reports including DM, hypercholesterolemia, MS, acne vulgaris, PCOS and menopausal hot flashes. To the best of our knowledge, omega-3 prevents the insulin resistance in DM through conversion to protectins and resolvins acting as an anti-inflammatory factor. Accordingly, unsaturated EFAs consumption such as EPO seems to be effective in DM [83]. Furthermore, hypercholesterolemia cause inflammation via leading to cell wall diseases, large artery endothelial dysfunction. As regards, unsaturated fatty acids improve the dysfunction by unclear mechanism, so as, consumption of fatty acids supplementation like EPO might be effective in this kind of disease [84].

Acne vulgaris, a disease with various skin lesions and increasing prevalence, can cause decreased self-confidence. Omega-6 supplementation like EPO cause effectiveness in patients by its antibacterial mechanism. Menopausal hot flashes can be reduced by PUFAs supplementation due to their effect on serotoninergic system, neuronal membrane and neurotransmitter function adjustment [85, 86]. Omega-6 fatty acids showed effectiveness in MS by experimental allergic encephalomyelitis suppression [87]. However, attention should be paid to conducting more studies and evaluate the possible drug-interaction of the supplement with the prescribed drugs.

The oral form of EPO that was administered in those studies were formulated in the form of soft gels, containing 71–72% LA and 7–9% GLA in combination with antioxidants like vitamin E or tocopherol, mostly under the brand names of Efamol or Epogam. The EPO content in each soft gel was in the range of 250, 500, 1000 or 1300 mg. One study that was included in this review utilized EPO in the topical formulation as seedcake for skin hydration where EPO was extracted by ethanol (50%, v/v). Elsewhere, in 2 other studies EPO was administered to the AE patients and healthy volunteers (evaluating the skin atrophy) in topical pharmaceutical formulation in types of O/W and W/O emulsion [49]. Also, oral liquid form of EPO in dark bottle, similar to Efamol manufacturer, was given to the patients with CF in one study [68]. Accordingly, EPO in the all types of formulations in the studied diseases showed varied effectiveness due to the type of disease and dosage of the EPO that was used.

Concisely, EPO seems to be profitable in inflammatory diseases especially on RA, AE, mastalgia, hypercholesterolemia, DM, MC, TD, menopausal hot flashes, acne vulgaris, MS. One of the limitations of this study was the exclusion of some studies that might have useful content due to the reasons mentioned in the text. The next important object was that not all factors were included in the measurement of the inflammatory factors. Nevertheless, the advantage that the studies had was that the EPO in question was mainly formulated orally or topically and was used, which is very convenient for patients, considering this feature, it is suggested that researchers in the future on the formulation of the oral form sublingual form is also concentrated considering that have high bioavailability and may reach a satisfactory treatment result faster. It is also suggested to increase the number of participants and use different ages and genders in clinical trial studies. (This study contains the following limitations:

- The mentioned studies in the text, have not assessed all the types of inflammatory factors.
- The main form of EPO in the trials was capsule and the patients had to consume high dose of EPO to understand the effect. So, the high number of capsules could lead to decrease the patients’ compliance. Consequently, loading more amounts of EPO in one capsule as a new formulation can be profitable in next studies.
- Increasing the number, genders and range of the age of participants can improve the quality of next studies.

## Conclusion

Overall, analyses of gathered data upon effectiveness of EPO supplementation in inflammatory diseases revealed that EPO publicized the most positive results in AE, mastalgia and RA, reducing disease symptoms and promoting improvements. Likewise, EPO was effective in DM, hypercholesterolemia, MS, acne vulgaris, PCOS and menopausal hot flashes in a few studies, However, we might not decisively claim its effectiveness in these diseases due to the low levels of evidences necessitating all-inclusive clinical trials. Nonetheless, it is recommended that future studies should be conducted to establish more comprehensive and generalizable results, further high-quality research is mandatory with larger sample sizes, longer interventions, with detailed investigation of influential inflammatory factors. Well-designed clinical trials combined with larger sample sizes and rigorous methodologies would provide a better understanding of the potential benefits and mechanisms of action for the EPO. These studies not only would be helping in determination of the appropriate dosage, treatment duration for any specific inflammatory diseases, but also could identify any potential side effects or interactions related to EPO with other medications.

### Supplementary Information

**Additional file 1.**

## Data Availability

All data generated or analysed during this study are included in this published article.

## References

[1] Steckel LE, Sosnoskie LM, Steckel SJ (2019). Common evening-primrose (Oenothera biennis L.). Weed Technology..

[2] Stonemetz D (2008). A review of the clinical efficacy of evening primrose. Holist Nurs Pract.

[3] Calder PC, Albers R, Antoine J-M, Blum S, Bourdet-Sicard R, Ferns G, Folkerts G, Friedmann P, Frost G, Guarner F (2009). Inflammatory disease processes and interactions with nutrition. Br J Nutr.

[4] Jima TT, Megersa M. Ethnobotanical study of medicinal plants used to treat human diseases in Berbere District, Bale Zone of Oromia Regional State, South East Ethiopia. Evidence-Based Complementary and Alternative Medicine. 2018;2018:8602945. 10.1155/2018/8602945 PMC6076952 30105073

[5] Shukla DA. Traditional medicines the way to lead life in current era. Int J Multidisip Res. 2020;9(12):135-39.

[6] Wiart C. Medicinal plants of the Asia-Pacific: drugs for the future? Boca Raton: Imprint CRC Press, World Scientific; 2006.

[7] Tasneem S, Liu B, Li B, Choudhary MI, Wang W (2019). Molecular pharmacology of inflammation: Medicinal plants as anti-inflammatory agents. Pharmacol Res.

[8] Daniyal M, Wang W. Molecular pharmacology of inflammation: Medicinal plants as antiinflammatory agents. In: Inflammation and Natural Products. Elsevier Science; 2021: p. 21–63.

[9] Fecker R, Buda V, Alexa E, Avram S, Pavel IZ, Muntean D, Cocan I, Watz C, Minda D, Dehelean CA (2020). Phytochemical and biological screening of Oenothera biennis L. hydroalcoholic extract. Biomolecules.

[10] Wettasinghe M, Shahidi F, Amarowicz R (2002). Identification and Quantification of Low Molecular Weight Phenolic Antioxidants in Seeds of Evening Primrose (Oenothera biennis L.). J Agric Food Chem..

[11] Shahidi F, Amarowicz R, He Y, Wettasinghe M (1997). Antioxidant Activity of Phenolic Extracts of Evening Primrose (Oenothera biennis): A Preliminary Study. J Food Lipids.

[12] Granica S, Czerwińska ME, Piwowarski JP, Ziaja M, Kiss AK (2013). Chemical composition, antioxidative and anti-inflammatory activity of extracts prepared from aerial parts of Oenothera biennis L. and Oenothera paradoxa Hudziok obtained after seeds cultivation. J Agric Food Chem..

[13] Khan K, Kunz R, Kleijnen J, Antes G. Systematic reviews to support evidence-based medicine. Hodder Education Publishers CRC press; 2011.

[14] Moher D, Liberati A, Tetzlaff J, Altman DG (2009). Group* P: Preferred reporting items for systematic reviews and meta-analyses: the PRISMA statement. Ann Intern Med.

[15] Veselinovic M, Vasiljevic D, Vucic V, Arsic A, Petrovic S, Tomic-Lucic A, Savic M, Zivanovic S, Stojic V, Jakovljevic V (2017). Clinical benefits of n-3 PUFA and ɤ-linolenic acid in patients with rheumatoid arthritis. Nutrients.

[16] Jamal G, Carmichael H (1990). The effect of γ-linolenic acid on human diabetic peripheral neuropathy: a double-blind placebo-controlled trial. Diabet Med.

[17] Arisaka M, Arisaka O, Yamashiro Y (1991). Fatty acid and prostaglandin metabolism in children with diabetes mellitus. II.—the effect of evening primrose oil supplementation on serum fatty acid and plasma prostaglandin levels. Prostaglandins, leukot essent fatty acids..

[18] Bamford JT, Gibson RW, Renier CM (1985). Atopic eczema unresponsive to evening primrose oil (linoleic and γ-linolenic acids). J Am Acad Dermatol.

[19] Manku M, Horrobin D, Morse N, Wright S, Burton J (1984). Essential fatty acids in the plasma phospholipids of patients with atopic eczema. Br J Dermatol.

[20] Wright S, Burton J (1982). Oral evening-primrose-seed oil improves atopic eczema. The Lancet.

[21] Berth-Jones J, Graham-Brown R (1993). Placebo-controlled trial of essential fatty acid supplementation in atopic dermatitis. The Lancet.

[22] Whitaker DK, Cilliers J, de Beer C (1996). Evening primrose oil (Epogam) in the treatment of chronic hand dermatitis: disappointing therapeutic results. Dermatology (Basel, Switzerland).

[23] Ebden P, Bevan C, Banks J, Fennerty A, Walters E (1989). A study of evening primrose seed oil in atopic asthma. Prostaglandins Leukot Essent Fatty Acids..

[24] Hederos C-A, Berg A (1996). Epogam evening primrose oil treatment in atopic dermatitis and asthma. Arch Dis Child.

[25] Blommers J, de Lange-de Klerk ES, Kuik DJ, Bezemer PD, Meijer S (2002). Evening primrose oil and fish oil for severe chronic astalgia: a randomized, double-blind, controlled trial. Am J Obstet Gynecol.

[26] Goyal A, Mansel RE (2005). A randomized multicenter study of gamolenic acid (Efamast) with and without antioxidant vitamins and minerals in the management of mastalgia. Breast J.

[27] Pye J, Mansel R, Hughes L (1985). Clinical experience of drug treatments for mastalgia. The Lancet.

[28] Qureshi S, Sultan N (2005). Topical nonsteroidal antiinflammatory drugs versus oil of evening primrose in the treatment of mastalgia. The Surgeon.

[29] Nasri K, Akrami S, Rahimi M, Taghizadeh M, Behfar M, Mazandaranian MR, Kheiry A, Memarzadeh MR, Asemi Z (2018). The effects of vitamin D and evening primrose oil co-supplementation on lipid profiles and biomarkers of oxidative stress in vitamin D-deficient women with polycystic ovary syndrome: A randomized, double-blind, placebo-controlled trial. Endocr Res.

[30] Farzaneh F, Fatehi S, Sohrabi M-R, Alizadeh K (2013). The effect of oral evening primrose oil on menopausal hot flashes: a randomized clinical trial. Arch Gynecol Obstet.

[31] Gateley C, Maddox P, Pritchard G, Sheridan W, Harrison B, Pye J, Webster D, Hughes L, Mansel R (1992). Plasma fatty acid profiles in benign breast disorders. Journal of British Surgery.

[32] Ishikawa T, Fujiyama Y, Igarashi O, Morino M, Tada N, Kagami A, Sakamoto T, Nagano M, Nakamura H (1989). Effects of gammalinolenic acid on plasma lipoproteins and apolipoproteins. Atherosclerosis.

[33] Jenkins A, Green A, Thompson R (1996). Essential fatty acid supplementation in chronic hepatitis B. Aliment Pharmacol Ther.

[34] Khoo SK, Munro C, Battistutta D (1990). Evening primrose oil and treatment of premenstrual syndrome. Med J Aust.

[35] Kokke KH, Morris JA, Lawrenson JG (2008). Oral omega-6 essential fatty acid treatment in contact lens associated dry eye. Cont Lens Anterior Eye.

[36] Laivuori H, Hovatta O, Viinikka L, Ylikorkala O (1993). Dietary supplementation with primrose oil or fish oil does not change urinary excretion of prostacyclin and thromboxane metabolites in pre-eclamptic women. Prostaglandins Leukot Essent Fatty Acids.

[37] Moodley J, Norman R (1989). Attempts at dietary alteration of prostaglandin pathways in the management of pre-eclampsia. Prostaglandins Leukot Essent Fatty Acids.

[38] Makrides M, Neumann MA, Simmer K, Gibson RA (1995). Erythrocyte fatty acids of term infants fed either breast milk, standard formula, or formula supplemented with long-chain polyunsaturates. Lipids.

[39] Manthorpe R, Petersen SH, Prause J (1984). Primary Sjögren's syndrome treated with Efamol/Efavit: A double-blind cross-over investigation. Rheumatol Int.

[40] Oliwiecki S, Burton J (1994). Evening primrose oil and marine oil in the treatment of psoriasis. Clin Exp Dermatol.

[41] Theander E, Horrobin DF, Jacobsson LT, Manthorpe R (2002). Gammalinolenic acid treatment of fatigue associated with primary Sjögren's syndrome. Scand J Rheumatol.

[42] Kaźmierska A, Bolesławska I, Polańska A, Dańczak-Pazdrowska A, Jagielski P, Drzymała-Czyż S, Adamski Z, Przysławski J (2022). Effect of Evening Primrose Oil Supplementation on Selected Parameters of Skin Condition in a Group of Patients Treated with Isotretinoin—A Randomized Double-Blind Trial. Nutrients.

[43] Oxholm P, Manthorpe R, Prause JU, Horrobin D (1986). Patients with primary Sjögren's syndrome treated for two months with evening primrose oil. Scand J Rheumatol.

[44] Rezapour-Firouzi S, Arefhosseini SR, Ebrahimi-Mamaghani M, Farhoudi M, Baradaran B, Ali TM, Zamani F (2013). Erythrocyte membrane fatty acids in multiple sclerosis patients and hot-nature dietary intervention with co-supplemented hemp-seed and evening-primrose oils. Afr J Tradit Complement Altern Med.

[45] Rezapour-Firouzi S, Arefhosseini SR, Ebrahimi-Mamaghani M, Baradaran B, Sadeghihokmabad E, Torbati M, Mostafaei S, Chehreh M, Zamani F (2014). Activity of liver enzymes in multiple sclerosis patients with Hot-nature diet and co-supplemented hemp seed, evening primrose oils intervention. Complement Ther Med.

[46] Rezapour-Firouzi S, Arefhosseini SR, Ebrahimi-Mamaghani M, Baradaran B, Sadeghihokmabad E, Mostafaei S, Torbati M, Chehreh M (2015). Alteration of delta-6-desaturase (FADS2), secretory phospholipase-A2 (sPLA2) enzymes by Hot-nature diet with co-supplemented hemp seed, evening primrose oils intervention in multiple sclerosis patients. Complement Ther Med.

[47] Vaddadi KS, Courtney P, Gilleard CJ, Manku MS, Horrobin DF (1989). A double-blind trial of essential fatty acid supplementation in patients with tardive dyskinesia. Psychiatry Res.

[48] Veale D, Torley H, Richards I (1994). O'DOWD A, Fttzsimons C, Belch J, Sturrock R: A double-blind placebo controlled trial of Efamol Marine on skin and joint symptoms of psoriatic arthritis. Rheumatology.

[49] Gehring W, Bopp R, Rippke F, Gloor M (1999). Effect of topically applied evening primrose oil on epidermal barrier function in atopic dermatitis as a function of vehicle. Arzneimittelforschung.

[50] Oliwiecki S, Armstrong J, Burton J, Bradfield J (1993). The effect of essential fatty acids on epidermal atrophy due to topical steroids. Clin Exp Dermatol.

[51] Ratz-Łyko A, Arct J, Pytkowska K, Majewski S (2015). In vivo and ex vivo evaluation of cosmetic properties of seedcakes. J Cosmet Laser Ther.

[52] Cumpston M, Li T, Page MJ, Chandler J, Welch VA, Higgins JP, Thomas J (2019). Updated guidance for trusted systematic reviews: a new edition of the Cochrane Handbook for Systematic Reviews of Interventions. Cochrane database syst rev..

[53] Joanna Briggs Institute Levels of Evidence and Grades of Recommendation Working Party. (2013). JBI Grades of Recommendation. [https://wiki.joannabriggs.org/display/MANUAL/Appendix+9.1+JBI+grades+of+recommendation].

[54] Tomic-Smiljanic M, Vasiljevic D, Lucic-Tomic A, Andjelkovic N, Jakovljevic V, Bolovich S, Veselinovic M (2019). Influence of different supplementation on platelet aggregation in patients with rheumatoid arthritis. Clin Rheumatol.

[55] Belch J, Ansell D, Madhok R (1988). O'dowd A, Sturrock R: Effects of altering dietary essential fatty acids on requirements for non-steroidal anti-inflammatory drugs in patients with rheumatoid arthritis: a double blind placebo controlled study. Ann Rheum Dis.

[56] Brzeski M, Madhok R, Capell H (1991). Evening primrose oil in patients with rheumatoid arthritis and side-effects of non-steroidal anti-inflammatory drugs. Rheumatology.

[57] Jäntti J, Nikkari T, Solakivi T, Vapaatalo H, Isomäki H (1989). Evening primrose oil in rheumatoid arthritis: changes in serum lipids and fatty acids. Ann Rheum Dis.

[58] Horrobin D (1989). Effects of evening primrose oil in rheumatoid arthritis. Ann Rheum Dis.

[59] Alam U, Asghar O, Azmi S, Malik RA (2014). General aspects of diabetes mellitus. Handb Clin Neurol.

[60] Businco L, Cantani A. Mast cell blockers and atopic eczema. In: Handbook of atopic eczema. Berlin, Heidelberg: Springer; 1991: p. 407–414.

[61] Rezapour-Firouzi S, Arefhosseini SR, Mehdi F, Mehrangiz E-M, Baradaran B, Sadeghihokmabad E, Mostafaei S, Fazljou SMB (2013). Torbati M-a, Sanaie S: Immunomodulatory and therapeutic effects of Hot-nature diet and co-supplemented hemp seed, evening primrose oils intervention in multiple sclerosis patients. Complement Ther Med.

[62] Mihm MC, Soter NA, Dvorak HF, Austen KF (1976). The structure of normal skin and the morphology of atopic eczema. J Investig Dermatol.

[63] Schalin-Karrila M, Mattila L, Jansen C, Uotila P (1987). Evening primrose oil in the treatment of atopic eczema: effect on clinical status, plasma phospholipid fatty acids and circulating blood prostaglandins. Br J Dermatol.

[64] Schäfer L, Kragballe K (1991). Supplementation with evening primrose oil in atopic dermatitis: effect on fatty acids in neutrophils and epidermis. Lipids.

[65] Fei JZ, Perruccio A, Ye Y, Gladman DD, Chandran V (2017). SAT0481 The relationship between the patient acceptable symptom state (PASS) and disease activity in patients with psoriatic arthritis (PSA).

[66] Rosenfield RL, Ehrmann DA (2016). The pathogenesis of polycystic ovary syndrome (PCOS): the hypothesis of PCOS as functional ovarian hyperandrogenism revisited. Endocr Rev.

[67] Davis PB (2006). Cystic fibrosis since 1938. Am J Respir Crit Care Med.

[68] Dodge JA, Custance JM, Goodchild MC, Laing SC, Vaughan M (1990). Paradoxical effects of essential fatty acid supplementation on lipid profiles and sweat electrolytes in cystic fibrosis. Br J Nutr.

[69] Freedman RR (2014). Menopausal hot flashes: mechanisms, endocrinology, treatment. J Steroid Biochem Mol Biol.

[70] Lok AS, Heathcote EJ, Hoofnagle JH (2001). Management of hepatitis B: 2000—summary of a workshop. Gastroenterology.

[71] Orasanu G, Ziouzenkova O, Devchand PR, Nehra V, Hamdy O, Horton ES, Plutzky J (2008). The PPARγ agonist pioglitazone represses inflammation in a PPARα-dependent manner in vitro and in vivo in mice. J Am Coll Cardiol.

[72] Dickerson LM, Mazyck PJ, Hunter MH (2003). Premenstrual syndrome. Am Fam Physician.

[73] Kojima T (2018). Contact lens-associated dry eye disease: recent advances worldwide and in Japan. Invest Ophthalmol Vis Sci..

[74] Milloy M, Wood E (2015). Withdrawal from methadone in US prisons: cruel and unusual?. The Lancet.

[75] Sharma N, Gupta A, Jha PK, Rajput P (2012). Mastalgia cured! Randomized trial comparing centchroman to evening primrose oil. Breast J.

[76] Mol BW, Roberts CT, Thangaratinam S, Magee LA, De Groot CJ, Hofmeyr GJ (2016). Pre-eclampsia. The Lancet.

[77] Dobson R, Giovannoni G (2019). Multiple sclerosis–a review. Eur J Neurol.

[78] Manthorpe R, Hagen Petersen S, Prause JU (1984). Primary Sjögren's syndrome treated with Efamol/Efavit. A double-blind cross-over investigation Rheumatol Int.

[79] Boehncke WH, Schön MP (2015). Psoriasis. Lancet (London, England).

[80] Horrobin D, Ells K, Morse-Fisher N, Manku M (1991). The effects of evening primrose oil, safflower oil and paraffin on plasma fatty acid levels in humans: choice of an appropriate placebo for clinical studies on primrose oil. Prostaglandins Leukot Essent Fatty Acids.

[81] Timoszuk M, Bielawska K, Skrzydlewska E (2018). Evening primrose (Oenothera biennis) biological activity dependent on chemical composition. Antioxidants.

[82] Ahmed MM, Rushdy EA, Azm DAA, Sabry RM, El Nahas HG (2021). Omega-3 Fatty Acids plus Vitamin E Cosupplementation versus Vitamin E in Fibrocystic Breast Patient with Mastalgia: A Randomized Controlled Trial. Open Access Macedonian Journal of Medical Sciences.

[83] Candidate SYP, Candidate JSP (2018). Nissa Mazzola PharmD C: Nutritional supplements for the prevention of diabetes mellitus and its complications. Journal of nutrition & intermediary metabolism.

[84] Goodfellow J, Bellamy MF, Ramsey MW, Jones CJ, Lewis MJ (2000). Dietary supplementation with marine omega-3 fatty acids improve systemic large artery endothelial function in subjects with hypercholesterolemia. J Am Coll Cardiol.

[85] Sanchez-Borrego R, von Schacky C, Osorio MJA, Llaneza P, Pinto X, Losa F, Navarro MC, Lubián D, Mendoza N (2017). Recommendations of the Spanish Menopause Society on the consumption of omega-3 polyunsaturated fatty acids by postmenopausal women. Maturitas.

[86] Kheirkhah M, Gholami R, Ghare-shiran SY, Abbasinia H (2016). Comparison of the effect of omega-3 fatty acids and perforan (Hypericum perforatum) on severity of premenstrual syndrome (PMS): A randomized trial. International Journal of Medical Research & Health Sciences.

[87] Stewart T, Bowling A (2005). Polyunsaturated fatty acid supplementation in MS. Int MS J.

