# Supplementary material for: The effect of Oenothera biennis (Evening primrose) oil on inflammatory diseases: a systematic review of clinical trials
Source: BMC Complement Med Ther. 2024 Feb 15;24:89. doi: 10.1186/s12906-024-04378-5 (PMC10867995; doi:10.1186/s12906-024-04378-5)
Supplement: Supplementary file 1 — Additional file 1. [file 12906_2024_4378_MOESM1_ESM.zip › appendix. A.docx]

**Appendix F. Cochrane Risk of Bias Tool**

Use the modified Cochrane Collaboration tool to assess risk of bias for randomized controlled trials. Bias is assessed as a judgment (high, low, or unclear) for individual elements from five domains (selection, performance, attrition, reporting, and other).

**AUB KQ1 Risk of Bias Assessment (Reference ID # )**

| **Domain** | **Description** | **High Risk of Bias** | **Low Risk of Bias** | **Unclear Risk of Bias** | **Reviewer Assessment** | **Reviewer Comments** |
| --- | --- | --- | --- | --- | --- | --- |
| *Selection bias*  ***Random sequence generation*** | Described the method used to generate the allocation sequence in  sufficient detail to  allow an assessment of whether it should  produce comparable groups | Selection bias (biased allocation to interventions) due to inadequate generation of a randomized sequence | Random sequence generation method should produce comparable groups | Not described in sufficient detail | **High**  **Low**  **Unclear** |  |
| *Selection bias*  ***Allocation concealment*** | Described the method used to conceal the allocation  sequence in  sufficient detail to determine whether intervention allocations could have been foreseen before or during enrollment | Selection bias (biased allocation to interventions) due to inadequate concealment of allocations prior to assignment | Intervention allocations  likely could not have been foreseen in before or during enrollment | Not described in sufficient detail | **High**  **Low**  **Unclear** |  |
| *Reporting bias*  ***Selective reporting*** | Stated how the possibility of selective outcome reporting was examined by the authors and what was found | Reporting bias  due to selective outcome reporting | Selective outcome  reporting bias not detected | Insufficient information to permit judgment† | **High**  **Low**  **Unclear** |  |

tudy's protocol, responses should be provided for each question/entry.

† It is likely that the majority of studies will fall into this category.

Assess each main or class of outcomes for each of the following. Indicate the specific outcome.

**AUB KQ1 Risk of Bias Assessment (Reference ID # )**

**Outcome:**

| **Domain** | **Description** | **High Risk of Bias** | **Low Risk of Bias** | **Unclear Risk of Bias** | **Reviewer Assessment** | **Reviewer Comments** |
| --- | --- | --- | --- | --- | --- | --- |
| *Performance bias*  ***Blinding (participants and personnel)*** | Described all measures used, if any, to blind study participants and personnel from knowledge of which intervention a participant received. Provided any information relating to whether the intended blinding was effective. | Performance bias due to knowledge of the allocated interventions by participants and personnel during the study. | Blinding was likely effective. | Not described in sufficient detail | **High**  **Low**  **Unclear** |  |
| *Detection bias*  ***Blinding (outcome assessment)*** | Described all measures used, if  any, to blind outcome assessors from knowledge of which intervention a participant received. Provided any information relating to whether the intended blinding was effective. | Detection bias due to knowledge of the allocated  interventions by outcome assessors. | Blinding was likely effective. | Not described in sufficient detail | **High**  **Low**  **Unclear** |  |
| *Attrition bias*  ***Incomplete outcome data*** | Described the completeness of outcome data for each main outcome, including  attrition and exclusions from the analysis. Stated whether attrition and exclusions were reported, the numbers in each intervention group (compared with total randomized participants), reasons for attrition/exclusions where reported. | Attrition bias due to amount, nature or handling of incomplete outcome data. | Handling of incomplete outcome data was complete and unlikely to have produced bias | Insufficient reporting of attrition/exclusi ons to permit  judgment (e.g., number randomized not stated, no reasons for missing data provided) | **High**  **Low**  **Unclear** |  |
