# Supplementary material for: The effect of Oenothera biennis (Evening primrose) oil on inflammatory diseases: a systematic review of clinical trials
Source: BMC Complement Med Ther. 2024 Feb 15;24:89. doi: 10.1186/s12906-024-04378-5 (PMC10867995; doi:10.1186/s12906-024-04378-5)
Supplement: Supplementary file 1 — Additional file 1. [file 12906_2024_4378_MOESM1_ESM.zip › appendix. B.docx]

**Table.s1: Risk of Bias assessment for Arefhosseini study**

| **Bias** | **Judgement** | **Support for judgment** |
| --- | --- | --- |
| Random sequence generation (selection bias) | Low risk | Quote: It was fully mentioned in the method section and its flowchart was also explained.  Comment：Randomization method mentioned. |
| Allocation concealment (selection bias) | Unclear risk | Comment: Not mentioned in the article. |
| Blinding of participants and personnel (performance bias) | Unclear risk | Comment: There is no mention in the article. |
| Blinding of outcome assessment (detection bias) | Unclear risk | Comment: Not mentioned in the article. |
| Incomplete outcome data addressed (attrition bias) | Low risk | Quote: It was fully mentioned in the results section.  Comment: There is mention in the article |
| Selective reporting (reporting bias) | Low risk | Quote: It is mentioned in the Acknowledgment section of the article |

**Table.s2: Risk of Bias assessment for Arisaka study**

| **Bias** | **Judgement** | **Support for judgment** |
| --- | --- | --- |
| Random sequence generation (selection bias) | Low risk | Quote: It was fully mentioned in the method section. |
| Allocation concealment (selection bias) | Unclear risk | Comment: Not mentioned in the article. |
| Blinding of participants and personnel (performance bias) | Unclear risk | Comment: There is no mention in the article. |
| Blinding of outcome assessment (detection bias) | Unclear risk | Comment: There is no mention in the article. |
| Incomplete outcome data addressed (attrition bias) | Low risk | Quote: It was fully mentioned in the results section.  Comment: There is mention in the article. |
| Selective reporting (reporting bias) | High risk | Quote: Nothing is mentioned in the article. |

**Table.s3: Risk of Bias assessment for Bamford study**

| **Bias** | **Judgement** | **Support for judgment** |
| --- | --- | --- |
| Random sequence generation (selection bias) | Low risk | Quote : It was fully mentioned in the method section  Comment：Randomization method mentioned. |
| Allocation concealment (selection bias) | Low risk | Comment: In the method section, it is mentioned that labels were attached to the drugs in order to prevent patients from forgetting and not being bothered, but it is not mentioned that the patients recognized the contents from the labels. The labels were not clear as to what they contained. |
| Blinding of participants and personnel (performance bias) | Unclear risk | Comment: There is no mention in the article. |
| Blinding of outcome assessment (detection bias) | Unclear risk | Comment: Not mentioned in the article. |
| Incomplete outcome data addressed (attrition bias) | Low risk | Quote: It was fully mentioned in the results section.  Comment: There is mention in the article |
| Selective reporting (reporting bias) | High risk | Comment: Nothing is mentioned in the article. |

**Table.s4: Risk of Bias assessment for Belch study**

| **Bias** | **Judgement** | **Support for judgment** |
| --- | --- | --- |
| Random sequence generation (selection bias) | Low risk | Quote: It was fully mentioned in the method section.  A full paragraph is written on the study design  Comment：Randomization method mentioned. |
| Allocation concealment (selection bias) | Low risk | Quote: In the text of the full article, it is mentioned that the study was double-blind and none of them had any knowledge, so it was completely concealed and it was not clear what they were consuming.  Comment: According to the mentioned cases, the low risk option was chosen. |
| Blinding of participants and personnel (performance bias) | unclear | Comment: There is no mention in the article. |
| Blinding of outcome assessment (detection bias) | unclear | Comment: Not mentioned in the article. |
| Incomplete outcome data addressed (attrition bias) | Low risk | Quote: In the section with the title of patients, the process and number of patients attending or leaving is fully explained.  Comment: There is mention in the article. |
| Selective reporting (reporting bias) | High risk | Comment: Nothing is mentioned in the article. |

**Table.s5: Risk of Bias assessment for Bloomers study**

| **Bias** | **Judgement** | **Support for judgment** |
| --- | --- | --- |
| Random sequence generation (selection bias) | Low risk | Quote: It is fully explained in the section titled Trial design, medication, and assessment.  Comment：Randomization method mentioned. |
| Allocation concealment (selection bias) | Unclear risk | Comment: Nothing is mentioned in the article. |
| Blinding of participants and personnel (performance bias) | Unclear risk | Comment: There is no mention in the article. |
| Blinding of outcome assessment (detection bias) | Unclear risk | Comment: Not mentioned in the article. |
| Incomplete outcome data addressed (attrition bias) | Unclear risk | Comment: There is no mention in the article. |
| Selective reporting (reporting bias) | High risk | Comment: Nothing is mentioned in the article. |

**Table.s6: Risk of Bias assessment for Berth-jones study**

| **Bias** | **Judgement** | **Support for judgment** |
| --- | --- | --- |
| Allocation concealment (selection bias) | unclear risk | Comment: There is no mention in the article. |
| Blinding of participants and personnel (performance bias) | Unclear risk | Comment: There is no mention in the article. |
| Blinding of outcome assessment (detection bias) | Low risk | Comment: They did not know the results. |
| Incomplete outcome data addressed (attrition bias) | Low risk | Quote: It is explained in the full results section.  Comment: There is mention in the article. |
| Selective reporting (reporting bias) | High risk | Comment: Nothing is mentioned in the article. |

**Table.s7: Risk of Bias assessment for Brzeski study**

| **Bias** | **Judgement** | **Support for judgment** |
| --- | --- | --- |
| Random sequence generation (selection bias) | Low risk | Quote: It was fully mentioned in the method section.  Comment：Randomization method mentioned. |
| Allocation concealment (selection bias) | \|Unclear risk | Comment: There is no mention in the article. |
| Blinding of participants and personnel (performance bias) | Unclear risk | Comment: There is no mention in the article. |
| Blinding of outcome assessment (detection bias) | Low risk | Comment: They did not know the results. |
| Incomplete outcome data addressed (attrition bias) | Low risk | Quote: It is explained in the full results section.  Comment: There is mention in the article. |
| Selective reporting (reporting bias) | High risk | Comment: Nothing is mentioned in the article. |

**Table.s8: Risk of Bias assessment for Ebden study**

| **Bias** | **Judgement** | **Support for judgment** |
| --- | --- | --- |
| Random sequence generation (selection bias) | Low risk | Quote: It was fully mentioned in the method section.  Comment：Randomization method mentioned. |
| Allocation concealment (selection bias) | unclear risk | Comment: There is no mention in the article. |
| Blinding of participants and personnel (performance bias) | unclear | Comment: There is no mention in the article. |
| Blinding of outcome assessment (detection bias) | Unclear risk | Comment: Nothing is mentioned in the article |
| Incomplete outcome data addressed (attrition bias) | Unclear risk | Comment: There is no mention in the article. |
| Selective reporting (reporting bias) | High risk | Comment: Nothing is mentioned in the article. |

**Table.s9: Risk of Bias assessment for Farzaneh study**

| **Bias** | **Judgement** | **Support for judgment** |
| --- | --- | --- |
| Random sequence generation (selection bias) | Low risk | Quote: It was fully mentioned in the method section.  Comment：Randomization method mentioned. |
| Allocation concealment (selection bias) | unclear risk | Comment: There is no mention in the article. |
| Blinding of participants and personnel (performance bias) | Unclear risk | Comment: There is no mention in the article. |
| Blinding of outcome assessment (detection bias) | Unclear risk | Comment: Nothing is mentioned in the article |
| Incomplete outcome data addressed (attrition bias) | Unclear risk | Comment: There is no mention in the article. |
| Selective reporting (reporting bias) | High risk | Comment: Nothing is mentioned in the article. |

**Table.s10: Risk of Bias assessment for Gateley study**

| **Bias** | **Judgement** | **Support for judgment** |
| --- | --- | --- |
| Random sequence generation (selection bias) | Low risk | Quote: It was fully mentioned in the method section.  Comment：Randomization method mentioned. |
| Allocation concealment (selection bias) | unclear risk | Comment: There is no mention in the article. |
| Blinding of participants and personnel (performance bias) | unclear | Comment: There is no mention in the article. |
| Blinding of outcome assessment (detection bias) | Unclear risk | Comment: Nothing is mentioned in the article |
| Incomplete outcome data addressed (attrition bias) | Unclear risk | Comment: There is no mention in the article. |
| Selective reporting (reporting bias) | High risk | Comment: Nothing is mentioned in the article. |

**Table.s11: Risk of Bias assessment for Gehring study**

| **Bias** | **Judgement** | **Support for judgment** |
| --- | --- | --- |
| Random sequence generation (selection bias) | Low risk | Quote: It was fully mentioned in the method section.  Comment：Randomization method mentioned. |
| Allocation concealment (selection bias) | unclear risk | Comment: There is no mention in the article. |
| Blinding of participants and personnel (performance bias) | High risk | Comment: It is mentioned in the text that they knew |
| Blinding of outcome assessment (detection bias) | High risk | Comment: It was not blinding at all. |
| Incomplete outcome data addressed (attrition bias) | Low risk | Comment: It is fully explained in the population section 1 , 2. |
| Selective reporting (reporting bias) | High risk | Comment: Nothing is mentioned in the article. |

**Table.s12: Risk of Bias assessment for Goyal study**

| **Bias** | **Judgement** | **Support for judgment** |
| --- | --- | --- |
| Random sequence generation (selection bias) | Low risk | Quote: It was fully mentioned in the method section.  Comment：Randomization method mentioned. |
| Allocation concealment (selection bias) | Unclear risk | Comment: There is no mention in the article. |
| Blinding of participants and personnel (performance bias) | Unclear | Comment: There is no mention in the article. |
| Blinding of outcome assessment (detection bias) | Unclear risk | Comment: Nothing is mentioned in the article |
| Incomplete outcome data addressed (attrition bias) | Low risk | Comment: It is fully explained in the text and the flowchart is fully drawn. |
| Selective reporting (reporting bias) | High risk | Comment: Nothing is mentioned in the article. |

**Table.s13: Risk of Bias assessment for Gupta study**

| **Bias** | **Judgement** | **Support for judgment** |
| --- | --- | --- |
| Random sequence generation (selection bias) | Low risk | Quote: It was fully mentioned in the method section.  Comment：Randomization method mentioned. |
| Allocation concealment (selection bias) | Unclear risk | Comment: There is no mention in the article. |
| Blinding of participants and personnel (performance bias) | High risk | Quote: In the method section, it is fully explained that the patients were aware of the steps of consumption and supplements and the process.  Comment: There is mention in the article. |
| Blinding of outcome assessment (detection bias) | Unclear risk | Comment: Nothing is mentioned in the article |
| Incomplete outcome data addressed (attrition bias) | Unclear risk | Comment: Nothing is mentioned in the article |
| Selective reporting (reporting bias) | High risk | Comment: Nothing was mentioned in the acknowledgment part of the article |

**Table.s14: Risk of Bias assessment for Hederos study**

| **Bias** | **Judgement** | **Support for judgment** |
| --- | --- | --- |
| Random sequence generation (selection bias) | Low risk | Quote: It was fully mentioned in the method section.  Comment：Randomization method mentioned. |
| Allocation concealment (selection bias) | unclear risk | Comment: There is no mention in the article. |
| Blinding of participants and personnel (performance bias) | Unclear risk | Comment: There is no mention in the article. |
| Blinding of outcome assessment (detection bias) | Unclear risk | Comment: Nothing is mentioned in the article |
| Incomplete outcome data addressed (attrition bias) | Unclear risk | Comment: Nothing is mentioned in the article |
| Selective reporting (reporting bias) | High risk | Comment: Nothing is mentioned in the article. |

**Table.s15: Risk of Bias assessment for Ishikawa study**

| **Bias** | **Judgement** | **Support for judgment** |
| --- | --- | --- |
| Random sequence generation (selection bias) | Low risk | Quote: It was fully mentioned in the method section.  Comment：Randomization method mentioned. |
| Allocation concealment (selection bias) | Unclear risk | Comment: There is no mention in the article. |
| Blinding of participants and personnel (performance bias) | Unclear risk | Comment: There is no mention in the article. |
| Blinding of outcome assessment (detection bias) | Unclear risk | Comment: Nothing is mentioned in the article |
| Incomplete outcome data addressed (attrition bias) | Unclear risk | Comment: Nothing is mentioned in the article |
| Selective reporting (reporting bias) | High risk | Comment: Nothing is mentioned in the article. |

**Table.s16: Risk of Bias assessment for jamal study**

| **Bias** | **Judgement** | **Support for judgment** |
| --- | --- | --- |
| Random sequence generation (selection bias) | Low risk | Quote: It was fully mentioned in the method section.  Comment：Randomization method mentioned. |
| Allocation concealment (selection bias) | unclear risk | Comment: There is no mention in the article. |
| Blinding of participants and personnel (performance bias) | Unclear risk | Comment: There is no mention in the article. |
| Blinding of outcome assessment (detection bias) | Unclear risk | Comment: Nothing is mentioned in the article |
| Incomplete outcome data addressed (attrition bias) | Unclear risk | Comment: Nothing is mentioned in the article |
| Selective reporting (reporting bias) | High risk | Comment: Nothing is mentioned in the article. |

**Table.s17: Risk of Bias assessment for Jantti study**

| **Bias** | **Judgement** | **Support for judgment** |
| --- | --- | --- |
| Random sequence generation (selection bias) | Low risk | Quote: It was fully mentioned in the method section.  Comment：Randomization method mentioned. |
| Allocation concealment (selection bias) | unclear risk | Comment: There is no mention in the article. |
| Blinding of participants and personnel (performance bias) | Unclear risk | Comment: There is no mention in the article. |
| Blinding of outcome assessment (detection bias) | Unclear risk | Comment: Nothing is mentioned in the article |
| Incomplete outcome data addressed (attrition bias) | Unclear risk | Comment: Nothing is mentioned in the article |
| Selective reporting (reporting bias) | High risk | Comment: Nothing is mentioned in the article. |

**Table.s18: Risk of Bias assessment for Jenkins study**

| **Bias** | **Judgement** | **Support for judgment** |
| --- | --- | --- |
| Random sequence generation (selection bias) | Low risk | Quote: It was fully mentioned in the method section.  Comment：Randomization method mentioned. |
| Allocation concealment (selection bias) | Unclear risk | Comment: There is no mention in the article. |
| Blinding of participants and personnel (performance bias) | Unclear risk | Comment: There is no mention in the article. |
| Blinding of outcome assessment (detection bias) | Unclear risk | Comment: Nothing is mentioned in the article |
| Incomplete outcome data addressed (attrition bias) | Unclear risk | Comment: Nothing is mentioned in the article |
| Selective reporting (reporting bias) | High risk | Comment: there is no mentioned in the article. |

**Table.s19: Risk of Bias assessment for Ka´zmierska study**

| **Bias** | **Judgement** | **Support for judgment** |
| --- | --- | --- |
| Random sequence generation (selection bias) | Low risk | Quote: It was fully mentioned in the method section.  Comment：Randomization method mentioned. |
| Allocation concealment (selection bias) | Unclear risk | Comment: There is no mention in the article. |
| Blinding of participants and personnel (performance bias) | Unclear risk | Comment: There is no mention in the article. |
| Blinding of outcome assessment (detection bias) | Unclear risk | Comment: Nothing is mentioned in the article |
| Incomplete outcome data addressed (attrition bias) | Unclear risk | Comment: Nothing is mentioned in the article |
| Selective reporting (reporting bias) | Low risk | Comment: There is mentioned in the article. |

**Table.s20: Risk of Bias assessment for khoo study**

| **Bias** | **Judgement** | **Support for judgment** |
| --- | --- | --- |
| Random sequence generation (selection bias) | Low risk | Quote: It was fully mentioned in the method section.  Comment：Randomization method mentioned. |
| Allocation concealment (selection bias) | Unclear risk | Comment: There is no mention in the article. |
| Blinding of participants and personnel (performance bias) | Unclear risk | Comment: There is no mention in the article. |
| Blinding of outcome assessment (detection bias) | Unclear risk | Comment: Nothing is mentioned in the article |
| Incomplete outcome data addressed (attrition bias) | Unclear risk | Comment: Nothing is mentioned in the article |
| Selective reporting (reporting bias) | High risk | Comment: Nothing is mentioned in the article |

**Table.s21: Risk of Bias assessment for Laivuori study**

| **Bias** | **Judgement** | **Support for judgment** |
| --- | --- | --- |
| Random sequence generation (selection bias) | Low risk | Quote: It was fully mentioned in the method section.  Comment：Randomization method mentioned. |
| Allocation concealment (selection bias) | Unclear risk | Comment: There is no mention in the article. |
| Blinding of participants and personnel (performance bias) | Unclear risk | Comment: There is no mention in the article. |
| Blinding of outcome assessment (detection bias) | Unclear risk | Comment: Nothing is mentioned in the article |
| Incomplete outcome data addressed (attrition bias) | Unclear risk | Comment: Nothing is mentioned in the article |
| Selective reporting (reporting bias) | High risk | Comment: There is no mentioned in the article. |

**Table.s22: Risk of Bias assessment for Lens study**

| **Bias** | **Judgement** | **Support for judgment** |
| --- | --- | --- |
| Random sequence generation (selection bias) | Low risk | Quote: It was fully mentioned in the method section.  Comment：Randomization method mentioned. |
| Allocation concealment (selection bias) | Unclear risk | Comment: There is no mention in the article. |
| Blinding of participants and personnel (performance bias) | Unclear risk | Comment: There is no mention in the article. |
| Blinding of outcome assessment (detection bias) | Unclear risk | Comment: Nothing is mentioned in the article |
| Incomplete outcome data addressed (attrition bias) | Unclear risk | Comment: Nothing is mentioned in the article |
| Selective reporting (reporting bias) | High risk | Comment: Nothing is mentioned in the article |

**Table.s23: Risk of Bias assessment for Makrides study**

| **Bias** | **Judgement** | **Support for judgment** |
| --- | --- | --- |
| Random sequence generation (selection bias) | Low risk | Quote: It was fully mentioned in the method section.  Comment：Randomization method mentioned. |
| Allocation concealment (selection bias) | Unclear risk | Comment: There is no mention in the article. |
| Blinding of participants and personnel (performance bias) | Low risk | Comment: The staff and patients were unaware |
| Blinding of outcome assessment (detection bias) | Unclear risk | Comment: Nothing is mentioned in the article |
| Incomplete outcome data addressed (attrition bias) | Unclear risk | Comment: Nothing is mentioned in the article |
| Selective reporting (reporting bias) | High risk | Comment: Nothing is mentioned in the article |

**Table.s24: Risk of Bias assessment for Manku study**

| **Bias** | **Judgement** | **Support for judgment** |
| --- | --- | --- |
| Random sequence generation (selection bias) | Low risk | Quote: It was fully mentioned in the method section.  Comment：Randomization method mentioned. |
| Allocation concealment (selection bias) | Unclear risk | Comment: There is no mention in the article. |
| Blinding of participants and personnel (performance bias) | Unclear risk | Comment: There is no mention in the article. |
| Blinding of outcome assessment (detection bias) | Unclear risk | Comment: Nothing is mentioned in the article |
| Incomplete outcome data addressed (attrition bias) | Unclear risk | Comment: Nothing is mentioned in the article |
| Selective reporting (reporting bias) | High risk | Comment: Nothing is mentioned in the article |

**Table.s25: Risk of Bias assessment for Manthrope study**

| **Bias** | **Judgement** | **Support for judgment** |
| --- | --- | --- |
| Random sequence generation (selection bias) | Low risk | Quote: It was fully mentioned in the method section.  Comment：Randomization method mentioned. |
| Allocation concealment (selection bias) | Unclear risk | Comment: There is no mention in the article. |
| Blinding of participants and personnel (performance bias) | Unclear risk | Comment: There is no mention in the article. |
| Blinding of outcome assessment (detection bias) | Unclear risk | Comment: Nothing is mentioned in the article |
| Incomplete outcome data addressed (attrition bias) | Unclear risk | Comment: Nothing is mentioned in the article |
| Selective reporting (reporting bias) | High risk | Comment: Nothing is mentioned in the article |

**Table.s26: Risk of Bias assessment for Moodley study**

| **Bias** | **Judgement** | **Support for judgment** |
| --- | --- | --- |
| Random sequence generation (selection bias) | Unclear risk | Comment：There is no mention in the article. |
| Allocation concealment (selection bias) | Unclear risk | Comment: There is no mention in the article. |
| Blinding of participants and personnel (performance bias) | Unclear risk | Comment: There is no mention in the article. |
| Blinding of outcome assessment (detection bias) | Unclear risk | Comment: Nothing is mentioned in the article |
| Incomplete outcome data addressed (attrition bias) | High risk | Comment: Nothing is mentioned in the article |
| Selective reporting (reporting bias) | Unclear risk | Comment: Nothing is mentioned in the article |

**Table.s27: Risk of Bias assessment for Nasri study**

| **Bias** | **Judgement** | **Support for judgment** |
| --- | --- | --- |
| Random sequence generation (selection bias) | Low risk | Quote: It was fully mentioned in the method section.  Comment：Randomization method mentioned. |
| Allocation concealment (selection bias) | Unclear risk | Comment: There is no mention in the article. |
| Blinding of participants and personnel (performance bias) | Unclear risk | Comment: There is no mention in the article. |
| Blinding of outcome assessment (detection bias) | Unclear risk | Comment: Nothing is mentioned in the article |
| Incomplete outcome data addressed (attrition bias) | Low risk | Comment: there is mentioned in the article. Both as a flowchart and as an explanation in the text |
| Selective reporting (reporting bias) | Low risk | Comment: there is mentioned in the article |

**Table.s28: Risk of Bias assessment for OLIWIF.CKl 1992 study**

| **Bias** | **Judgement** | **Support for judgment** |
| --- | --- | --- |
| Random sequence generation (selection bias) | Low risk | Quote: It was fully mentioned in the method section.  Comment：Randomization method mentioned. |
| Allocation concealment (selection bias) | Unclear risk | Comment: There is no mention in the article. |
| Blinding of participants and personnel (performance bias) | Unclear risk | Comment: There is no mention in the article. |
| Blinding of outcome assessment (detection bias) | Unclear risk | Comment: Nothing is mentioned in the article |
| Incomplete outcome data addressed (attrition bias) | Unclear risk | Comment: there is mentioned in the article. Both as a flowchart and as an explanation in the text |
| Selective reporting (reporting bias) | High risk | Comment: there is not mentioned in the article |

**Table.s29: Risk of Bias assessment for OLIWIF.CKl 1993 study**

| **Bias** | **Judgement** | **Support for judgment** |
| --- | --- | --- |
| Random sequence generation (selection bias) | Low risk | Quote: It was fully mentioned in the method section.  Comment：Randomization method mentioned. |
| Allocation concealment (selection bias) | Unclear risk | Comment: There is no mention in the article. |
| Blinding of participants and personnel (performance bias) | Unclear risk | Comment: There is no mention in the article. |
| Blinding of outcome assessment (detection bias) | Unclear risk | Comment: Nothing is mentioned in the article |
| Incomplete outcome data addressed (attrition bias) | Unclear risk | Comment: there is mentioned in the article. Both as a flowchart and as an explanation in the text |
| Selective reporting (reporting bias) | High risk | Comment: there is not mentioned in the article |

**Table.s30: Risk of Bias assessment for Oxolm study**

| **Bias** | **Judgement** | **Support for judgment** |
| --- | --- | --- |
| Random sequence generation (selection bias) | Low risk | Quote: It was fully mentioned in the method section.  Comment：Randomization method mentioned. |
| Allocation concealment (selection bias) | Unclear risk | Comment: There is no mention in the article. |
| Blinding of participants and personnel (performance bias) | Unclear risk | Comment: There is no mention in the article. |
| Blinding of outcome assessment (detection bias) | Unclear risk | Comment: Nothing is mentioned in the article |
| Incomplete outcome data addressed (attrition bias) | Unclear risk | Comment: there is mentioned in the article. Both as a flowchart and as an explanation in the text |
| Selective reporting (reporting bias) | High risk | Comment: there is not mentioned in the article |

**Table.s31: Risk of Bias assessment for Pye study**

| **Bias** | **Judgement** | **Support for judgment** |
| --- | --- | --- |
| Random sequence generation (selection bias) | Low risk | Quote: It was fully mentioned in the method section.  Comment：Randomization method mentioned. |
| Allocation concealment (selection bias) | Unclear risk | Comment: There is no mention in the article. |
| Blinding of participants and personnel (performance bias) | Unclear risk | Comment: There is no mention in the article. |
| Blinding of outcome assessment (detection bias) | Unclear risk | Comment: Nothing is mentioned in the article |
| Incomplete outcome data addressed (attrition bias) | Unclear risk | Comment: there is mentioned in the article. Both as a flowchart and as an explanation in the text |
| Selective reporting (reporting bias) | High risk | Comment: there is not mentioned in the article |

**Table.s32: Risk of Bias assessment for Qureshi study**

| **Bias** | **Judgement** | **Support for judgment** |
| --- | --- | --- |
| Random sequence generation (selection bias) | High risk | Quote : There is no mention in the method part of the article  Comment：Randomization method is not mentioned. |
| Allocation concealment (selection bias) | Unclear risk | Comment: There is no mention in the article. |
| Blinding of participants and personnel (performance bias) | Unclear risk | Comment: There is no mention in the article. |
| Blinding of outcome assessment (detection bias) | Unclear risk | Comment: Nothing is mentioned in the article |
| Incomplete outcome data addressed (attrition bias) | Unclear risk | Comment: there is mentioned in the article. Both as a flowchart and as an explanation in the text |
| Selective reporting (reporting bias) | High risk | Comment: there is not mentioned in the article |

**Table.s33: Risk of Bias assessment for Ratz-Łyko study**

| **Bias** | **Judgement** | **Support for judgment** |
| --- | --- | --- |
| Random sequence generation (selection bias) | Low risk | Quote: It was fully mentioned in the method section.  Comment：Randomization method mentioned. |
| Allocation concealment (selection bias) | Unclear risk | Comment: There is no mention in the article. |
| Blinding of participants and personnel (performance bias) | Unclear risk | Comment: There is no mention in the article. |
| Blinding of outcome assessment (detection bias) | Unclear risk | Comment: Nothing is mentioned in the article |
| Incomplete outcome data addressed (attrition bias) | Unclear risk | Comment: there is mentioned in the article. Both as a flowchart and as an explanation in the text |
| Selective reporting (reporting bias) | High risk | Comment: there is not mentioned in the article |

**Table.s34: Risk of Bias assessment for Rezapour-Firouzi2013study**

| **Bias** | **Judgement** | **Support for judgment** |
| --- | --- | --- |
| Random sequence generation (selection bias) | Low risk | Quote: It was fully mentioned in the method section.  Comment：Randomization method mentioned. |
| Allocation concealment (selection bias) | Unclear risk | Comment: There is no mention in the article. |
| Blinding of participants and personnel (performance bias) | Low risk | Comment: They were completely blind |
| Blinding of outcome assessment (detection bias) | Low risk | Comment: They were completely blind |
| Incomplete outcome data addressed (attrition bias) | Low risk | Comment: there is mentioned in the article. Both as a flowchart and as an explanation in the text |
| Selective reporting (reporting bias) | High risk | Comment: there is not mentioned in the article |

**Table.s35: Risk of Bias assessment for Rezapour-Firouzi2014study**

| **Bias** | **Judgement** | **Support for judgment** |
| --- | --- | --- |
| Random sequence generation (selection bias) | Low risk | Quote: It was fully mentioned in the method section.  Comment：Randomization method mentioned. |
| Allocation concealment (selection bias) | Unclear risk | Comment: There is no mention in the article. |
| Blinding of participants and personnel (performance bias) | Unclear risk | Comment: There is no mention in the article. |
| Blinding of outcome assessment (detection bias) | Unclear risk | Comment: Nothing is mentioned in the article |
| Incomplete outcome data addressed (attrition bias) | Low risk | Comment: there is mentioned in the article. Both as a flowchart and as an explanation in the text |
| Selective reporting (reporting bias) | Low risk | Comment: It is written in full in the Acknowledgment section |

**Table.s36: Risk of Bias assessment for Rezapour-Firouzi2015study**

| **Bias** | **Judgement** | **Support for judgment** |
| --- | --- | --- |
| Random sequence generation (selection bias) | Low risk | Quote: It was fully mentioned in the method section.  Comment：Randomization method mentioned. |
| Allocation concealment (selection bias) | Unclear risk | Comment: There is no mention in the article. |
| Blinding of participants and personnel (performance bias) | Unclear risk | Comment: There is no mention in the article. |
| Blinding of outcome assessment (detection bias) | Unclear risk | Comment: Nothing is mentioned in the article |
| Incomplete outcome data addressed (attrition bias) | Low risk | Comment: there is mentioned in the article. Both as a flowchart and as an explanation in the text |
| Selective reporting (reporting bias) | Low risk | Comment: It is written in full in the Acknowledgment section |

**Table.s37: Risk of Bias assessment for SCHALIN-KARRILA study**

| **Bias** | **Judgement** | **Support for judgment** |
| --- | --- | --- |
| Random sequence generation (selection bias) | Low risk | Quote: It was fully mentioned in the method section.  Comment：Randomization method mentioned. |
| Allocation concealment (selection bias) | Unclear risk | Comment: There is no mention in the article. |
| Blinding of participants and personnel (performance bias) | Unclear risk | Comment: There is no mention in the article. |
| Blinding of outcome assessment (detection bias) | Unclear risk | Comment: Nothing is mentioned in the article |
| Incomplete outcome data addressed (attrition bias) | Low risk | Comment: there is mentioned in the article. Both as a flowchart and as an explanation in the text |
| Selective reporting (reporting bias) | Low risk | Comment: It is written in full in the Acknowledgment section |

**Table.s38: Risk of Bias assessment for Theander study**

| **Bias** | **Judgement** | **Support for judgment** |
| --- | --- | --- |
| Random sequence generation (selection bias) | Low risk | Quote: It was fully mentioned in the method section.  Comment：Randomization method mentioned. |
| Allocation concealment (selection bias) | Unclear risk | Comment: There is no mention in the article. |
| Blinding of participants and personnel (performance bias) | Unclear risk | Comment: There is no mention in the article. |
| Blinding of outcome assessment (detection bias) | Unclear risk | Comment: Nothing is mentioned in the article |
| Incomplete outcome data addressed (attrition bias) | Unclear risk | Comment: there is not mentioned in the article. |
| Selective reporting (reporting bias) | High risk | Comment: there is not mentioned in the Acknowledgment section |

**Table.s39: Risk of Bias assessment for Tomic-Smiljanic study**

| **Bias** | **Judgement** | **Support for judgment** |
| --- | --- | --- |
| Random sequence generation (selection bias) | Low risk | Quote: It was fully mentioned in the method section.  Comment：Randomization method mentioned. |
| Allocation concealment (selection bias) | Unclear risk | Comment: There is no mention in the article. |
| Blinding of participants and personnel (performance bias) | Unclear risk | Comment: There is no mention in the article. |
| Blinding of outcome assessment (detection bias) | Low risk | Comment: there is mentioned in the article |
| Incomplete outcome data addressed (attrition bias) | Low risk | Comment: there is mentioned in the article. Both as a flowchart and as an explanation in the text |
| Selective reporting (reporting bias) | Unclear risk | Comment: It is written in full in the Acknowledgment section |

**Table.s40:. Risk of Bias assessment for vaddadi study**

| **Bias** | **Judgement** | **Support for judgment** |
| --- | --- | --- |
| Random sequence generation (selection bias) | Low risk | Quote: It was fully mentioned in the method section.  Comment：Randomization method mentioned. |
| Allocation concealment (selection bias) | Unclear risk | Comment: There is no mention in the article. |
| Blinding of participants and personnel (performance bias) | Unclear risk | Comment: There is no mention in the article. |
| Blinding of outcome assessment (detection bias) | Unclear risk | Comment: Nothing is mentioned in the article |
| Incomplete outcome data addressed (attrition bias) | Unclear risk | Comment: there is not mentioned in the article. |
| Selective reporting (reporting bias) | High risk | Comment: there is not mentioned in the Acknowledgment section |

**Table.s41: Risk of Bias assessment for veala study**

| **Bias** | **Judgement** | **Support for judgment** |
| --- | --- | --- |
| Random sequence generation (selection bias) | Low risk | Quote: It was fully mentioned in the method section.  Comment：Randomization method mentioned. |
| Allocation concealment (selection bias) | Unclear risk | Comment: There is no mention in the article. |
| Blinding of participants and personnel (performance bias) | Unclear risk | Comment: There is no mention in the article. |
| Blinding of outcome assessment (detection bias) | Low risk | Comment: there is mentioned in the article |
| Incomplete outcome data addressed (attrition bias) | Low risk | Comment: there is mentioned in the article. Both as a flowchart and as an explanation in the text |
| Selective reporting (reporting bias) | High risk | Comment: there is not mentioned in the Acknowledgment section |

**Table.s42: Risk of Bias assessment for Veselinovic study**

| **Bias** | **Judgement** | **Support for judgment** |
| --- | --- | --- |
| Random sequence generation (selection bias) | Low risk | Quote: It was fully mentioned in the method section.  Comment：Randomization method mentioned. |
| Allocation concealment (selection bias) | Unclear risk | Comment: There is no mention in the article. |
| Blinding of participants and personnel (performance bias) | Unclear risk | Comment: There is no mention in the article. |
| Blinding of outcome assessment (detection bias) | Low risk | Comment: there is mentioned in the article |
| Incomplete outcome data addressed (attrition bias) | Low risk | Comment: there is mentioned in the article. Both as a flowchart and as an explanation in the text |
| Selective reporting (reporting bias) | Low risk | Comment: there is mentioned in the Acknowledgment section |

**Table.s43: Risk of Bias assessment for Whitaker study**

| **Bias** | **Judgement** | **Support for judgment** |
| --- | --- | --- |
| Random sequence generation (selection bias) | Low risk | Quote: It was fully mentioned in the method section.  Comment：Randomization method mentioned. |
| Allocation concealment (selection bias) | Unclear risk | Comment: There is no mention in the article. |
| Blinding of participants and personnel (performance bias) | Unclear risk | Comment: There is no mention in the article. |
| Blinding of outcome assessment (detection bias) | Unclear risk | Comment: there is no mentioned in the article |
| Incomplete outcome data addressed (attrition bias) | Unclear risk | Comment: there is not mentioned in the article. |
| Selective reporting (reporting bias) | High risk | Comment: there is not mentioned in the Acknowledgment section |

**Table.s44: Risk of Bias assessment for Wright study**

| **Bias** | **Judgement** | **Support for judgment** |
| --- | --- | --- |
| Random sequence generation (selection bias) | Low risk | Quote: It was fully mentioned in the method section.  Comment：Randomization method mentioned. |
| Allocation concealment (selection bias) | Unclear risk | Comment: There is no mention in the article. |
| Blinding of participants and personnel (performance bias) | Unclear risk | Comment: There is no mention in the article. |
| Blinding of outcome assessment (detection bias) | Unclear risk | Comment: there is no mentioned in the article |
| Incomplete outcome data addressed (attrition bias) | Unclear risk | Comment: there is mentioned in the article. Both as a flowchart and as an explanation in the text |
| Selective reporting (reporting bias) | High risk | Comment: there is not mentioned in the Acknowledgment section |
