# Supplementary material for: The effect of Oenothera biennis (Evening primrose) oil on inflammatory diseases: a systematic review of clinical trials
Source: BMC Complement Med Ther. 2024 Feb 15;24:89. doi: 10.1186/s12906-024-04378-5 (PMC10867995; doi:10.1186/s12906-024-04378-5)
Supplement: Supplementary file 1 — Additional file 1. [file 12906_2024_4378_MOESM1_ESM.zip › appendix. C.pdf]

JBI Grades of Recommendation

Developed by the Joanna Briggs Institute Levels of Evidence and Grades of Recommendation Working Party October 2013

JBI Grades of Recommendation	
Grade A	A 'strong' recommendation for a certain health management strategy where (1) it is clear that desirable effects outweigh undesirable effects of the strategy; (2) where there is evidence of adequate quality supporting its use; (3) there is a benefit or no impact on resource use, and (4) values, preferences and the patient experience have been taken into account.
Grade B	A 'weak' recommendation for a certain health management strategy where (1) desirable effects appear to outweigh undesirable effects of the strategy, although this is not as clear; (2) where there is evidence supporting its use, although this may not be of high quality; (3) there is a benefit, no impact or minimal impact on resource use, and (4) values, preferences and the patient experience may or may not have been taken into account.

The FAME (Feasibility, Appropriateness, Meaningfulness and Effectiveness) scale may help inform the wording and strength of a recommendation.

F – Feasibility; specifically:

- What is the cost effectiveness of the practice?
- Is the resource/practice available?
- Is there sufficient experience/levels of competency available?

A – Appropriateness; specifically:

- Is it culturally acceptable?
- Is it transferable/applicable to the majority of the population?
- Is it easily adaptable to a variety of circumstances?

M – Meaningfulness; specifically:

- Is it associated with positive experiences?
- Is it not associated with negative experiences?

E – Effectiveness; specifically:

- Was there a beneficial effect?
- Is it safe? (i.e is there a lack of harm associated with the practice?
